# Different sex determination systems in two closely related Eurasian minnow (*Phoxinus*) species

**DOI:** 10.1038/s41437-026-00827-8

**Published:** 2026-02-24

**Authors:** Temitope Opeyemi Oriowo, Sophie Helen Smith, Jana Thorman, Nils Sternberg, Astrid Böhne, Madlen Stange

**Affiliations:** Leibniz Institute for the Analysis of Biodiversity Change, Museum Koenig Bonn, Bonn, Germany

**Keywords:** Evolutionary genetics, Population genetics, Comparative genomics

## Abstract

Sex determination systems in teleost fishes are highly diverse, and even closely related species often evolve different mechanisms. Although hybridisation among Eurasian minnows (*Phoxinus*: Cypriniformes, Leuciscidae) is well documented, their sex determination systems remain unexplored. Here, we investigated the genetic basis of sex determination in *Phoxinus phoxinus* and *Phoxinus csikii* using whole-genome sequencing, with a combination of coverage, SNP-, and k-mer-based approaches to identify sex-associated genomic regions. Whole-genome coverage analyses revealed no chromosomes with significantly sex-biased coverage, consistent with homomorphic sex chromosomes in both species. In *P. phoxinus*, male-specific heterozygosity at sex-linked SNPs showed genotypic differences within two regions, namely on chromosomes 3 and 12. Analysis of SNPs in these regions revealed drainage-specific, sex-associated patterns, indicating the presence of population-specific sex-associated genomic loci and a male heterogametic (XX/XY) system in this species. In contrast, *P. csikii* females displayed unique genotypic differences in a different part of chromosome 3, pointing to a female heterogametic (ZZ/ZW) system. We further observed overrepresentation of male-specific DNA sequences in *P. phoxinus* and female-specific sequences in *P. csikii*, providing additional evidence for the presence of sex-specific genomic regions and differing sex determination mechanisms in both species. These results provide evidence that two closely related *Phoxinus* species possess distinct sex determination systems, which may contribute to reproductive isolation.

## Introduction

Sex determination is the process that initiates the developmental pathway of an organism towards a male or female phenotype (Capel 2017). In vertebrates, sex determination is governed or influenced by a variety of environmental and/or genetic mechanisms (Stöck et al. 2021). When sex chromosomes genotypically determine sex, species can be classified according to which sex is heterogametic, that is, the sex that produces two different types of gametes with respect to sex chromosomal content. In XX/XY systems, males are heterogametic (XY; producing both X- and Y-bearing sperm), while females are homogametic (XX; only X-bearing ova). In ZZ/ZW systems, females are heterogametic (ZW; with both Z- and W-bearing ova), while males are homogametic (ZZ; only Z-bearing sperm).

Sex chromosomes originate from a pair of autosomes that acquire a sex-determining gene (Meisel 2020). Recombination between the emerging sex chromosomes is suppressed near the sex-determining locus, which can help maintain linkage with traits under sex-specific or sexually antagonistic selection (Ponnikas et al. 2018). Other mechanisms, including structural changes, drift, or regulatory constraints may also contribute to the early stages of sex chromosome differentiation (D. Charlesworth 2017; Yuan et al. 2018). Over time, this suppression can expand across the sex chromosome, leading to the accumulation of deleterious mutations, repetitive DNA, and gene loss on the sex-limited chromosome (Y or W), resulting in its progressive degeneration and leading to heteromorphic sex chromosomes, i.e., sex chromosome pairs that differ in size and morphology and can be distinguished cytogenetically (Charlesworth et al. 2000).

XY- and ZW-sex determining systems are the most widespread genotypic sex determination systems among vertebrates and are highly conserved in mammals and birds: most mammals share the same XY system, while birds rely on a ZW system (Gamble and Zarkower 2012). Other vertebrate lineages have evolved more complex arrangements, among them teleost fishes (Kitano et al. 2024). Some species show structurally complex sex chromosomes that arise through chromosomal fusions e.g., in three-spine sticklebacks (*Gasterosteus aculeatus)* (Liu et al. 2022). Some species, such as *Leporinus elongatus*, display multiple systems. For example, they have a Z₁Z₁Z₂Z₂/Z₁W₁Z₂W₂ system, whereby both males and females carry a combination of sex chromosomes (Parise-Maltempi et al. 2007). Other species exhibit genotypic combinations that result from interactions among multiple sex-determining loci rather than from fusions, as found in *Metriaclima pyrsonotus*, where the female-specific W allele can override the effect of the male-determining Y chromosome, resulting in fish with a ZWXY genotype system developing as females (Ser et al. 2010). These examples highlight the diversity and evolutionary flexibility of vertebrate sex determination.

### Sex determination in teleost fishes

Teleost fishes exhibit a remarkable diversity of sex determination systems, ranging from purely genetic mechanisms to complex interactions between genetic and environmental factors, including frequent turnovers between XY and ZW sex chromosome systems, reflecting their extensive evolutionary diversity (Kitano et al. 2024). Heteromorphic sex chromosomes are rare, suggesting either recent sex chromosome origins, incomplete recombination suppression or sustained long-term homomorphy (Schartl 2004). This can complicate the detection of sex chromosomes, which often requires high-resolution sequencing rather than karyotypic analysis (Palmer et al. 2019). Further complexity arises from the influence of environmental factors, such as temperature, also on species that depend on genetic sex determination.

In some species, sex determination can be driven by a single master sex-determining gene, which act as pivotal switch initiating the male developmental pathway, such as *dmrt1*, identified in several species, including *Oryzias latipes* (Nanda et al. 2002), and *sdY*, found in most salmonids (Yann et al. 2018). Sometimes Y-specific paralogs of autosomal genes with a role in sexual development (e.g. *amhy*, *dmy*) evolve, acquiring novel sex-determining functions (Kitano et al. 2024). However, this single-locus model does not apply universally. In species such as the cichlid *Astatotilapia burtoni*, sex determination is polygenic and involves multiple independently segregating loci (Lichilín et al. 2023). Polygenic sex determination allows for greater developmental flexibility and may help maintain genetic variation in sex-determining pathways (Moore et al. 2022); however, such polygenic sex determination may in fact represent a transient snapshot of the process of sex chromosome turnover (Schartl et al. 2023). The diversity of sex-determining mechanisms makes teleost fishes a relevant group for studying sex chromosome evolution, reproductive barriers, and speciation.

### Differences in sex determination in closely related species

It is not uncommon for closely related species to exhibit both male and female heterogamety (Brykov 2014), although female‑heterogametic (ZW) systems appear to be comparatively rare in fishes (Sember et al. 2021). Both systems frequently arise through sex chromosome turnover driven by the emergence of new sex-determining loci or chromosomal rearrangements (Furman et al. 2020). Turnover of sex chromosomes can be promoted by hybridization or sexual conflict (Pennell et al. 2018). This diversity is shaped by both allopatric and sympatric speciation. In allopatry, geographic isolation allows populations to accumulate genetic changes in sex determination independently through drift or local adaptation (Dufresnes and Crochet 2022). In sympatry, hybridization between diverged populations can reveal incompatibilities, such as hybrid sterility or skewed sex ratios, thereby creating strong selection for novel or reinforced sex-determining mechanisms. This has been observed, for example, in two cricket species, *Teleogyllus commodus* and *Teleogyllus oceanicus*, where X-linked divergence suggests that sex-linked genotypic differentiation contributes to the maintenance of species boundaries (Moran et al. 2018). A similar pattern is seen in sympatric three-spine sticklebacks, where a chromosomal fusion created a neo-sex chromosome in the Japan Sea lineage, contributing to phenotypic divergence and reproductive isolation from the Pacific Ocean lineage (Yoshida et al. 2014). Thus, divergence in isolation combined with evolutionary responses to hybrid conflict can drive rapid changes, possibly including switches in heterogamety in closely related species that may in turn facilitate or reinforce speciation (Ravinet et al. 2017).

Changes in heterogamety have been observed in vertebrates, where transitions occur between XY and ZW systems, e.g. in reptiles such as snakes (Pšenička et al. 2025), and amphibians, notably in frogs (Roco et al. 2015). Sex chromosome turnover appears also in teleost fish (El Taher et al. 2021; Wang et al. 2024), likely because many lineages have poorly differentiated sex chromosomes, so the X or Z chromosome can revert to being an autosome if a new sex-determining locus evolves, while the degenerated Y or W chromosome is lost (Bachtrog et al. 2014).

In addition, the rapid speciation rates in teleosts (Rabosky 2020), create repeated opportunities for novel sex-determining mutations to arise and become fixed, leading to a higher observed rate of sex-chromosome turnovers than in other vertebrate groups (Blaser et al. 2013). A well-researched example of sex chromosome turnover in closely related teleost fish species is the cichlids of Lake Tanganyika (El Taher et al. 2021). Further reports exist for Tilapiine cichlids, where *Oreochromis karongae* and *Tilapia mariae* exhibit female heterogamety, while their close relatives *Oreochromis niloticus* and *Oreochromis mossambicus* display male heterogamety (Cnaani et al. 2008). The same variation in heterogamety has been observed in *Oryzias* species (Takehana et al. 2008) as well as sticklebacks (Ross et al. 2009). These examples demonstrate the high lability of sex determination systems among closely related teleosts and raises questions about the frequency of such transitions in other freshwater species, e.g. in Eurasian minnows (*Phoxinus*: Cypriniformes, Leuciscidae)—a species complex distributed across Eurasian freshwater habitats (Palandačić et al. 2015).

In this study, we used the *Phoxinus* system to study the genotypic basis of sex determination in two relatively young (Palandačić et al. 2020) and closely related species to further our understanding of sex chromosome systems and turnover thereof in freshwater teleosts fishes.

### Sex determination in two closely related *Phoxinus* species

Eurasian minnows are small gonochoristic (Rasotto et al. 1987) freshwater fishes, which are a valuable case for investigating sex-determining systems among closely related species due to their wide geographical distribution, frequent sympatry, and unresolved species boundaries (Kottelat and Freyhof 2007). Its members occupy overlapping ranges, hybridise and inhabit diverse ecological settings (Palandačić et al. 2017, 2024), conditions that can promote diversification in sex determination and create hybrid conflict.

The two focal species of this study *P. phoxinus* (Linnaeus 1758), and *P. csikii* (Hankó 1922) occur in close geographic proximity, within major central European river basins (Palandačić et al. 2015, 2017; Denys et al. 2020). *P. phoxinus* is primarily distributed in the Meuse and Rhine drainages, whereas *P. csikii* is centred in the Danube and Rhine drainages (Palandačić et al. 2015, 2017; Denys et al. 2020). A potential hybrid zone between the two species has been reported at the Middle-Lower Rhine boundary (Denys et al. 2020; Sternberg et al. 2025). Both speciation and secondary contact of the two species could potentially be established by the shift of ancient rivers between major European freshwater basins, as recently described for the genus *Telestes* (Buj et al. 2017). Hybridization involving other *Phoxinus* species is documented between *P. phoxinus, P. csikii, P. septimaniae*, and *P. morella* (Palandačić et al. 2017) and arises both from natural contact zones and human-mediated introduction (Esposito et al. 2024; Sternberg et al. 2025).This geographic context makes it relevant to examine whether the species differ in their sex determination systems. Understanding the genotypic basis of sex determination could inform assessments of the potential risks associated with ongoing or future hybridisation in *Phoxinus*. These risks include reduced viability from genetic incompatibilities and potentially genetic swamping (Todesco et al. 2016).

To date, our understanding of sex determination in this genus remains limited due to taxonomic uncertainties and a previous lack of genomic resources. In this study, we aimed to identify sex-linked loci within each species and to determine whether these two species share the same sex determination system.

To achieve this, we analysed *P. phoxinus* from Meuse and Lower/Middle Rhine boundary and *P. csikii* from the Danube and Neckar (draining into the Upper Rhine). We first assembled partial mitogenomes to reconstruct phylogenetic relationships. Our aim was to confirm species identity and ensure the correct taxonomic classification of the specimens included in this study. We then applied a combination of complementary approaches that enable us to detect regions of differentiation between male and female genomes, thus helping to identify potential sex chromosomes. We applied (i) a coverage-based analysis to detect sex-specific copy number differences that would result from degeneration of the sex-limited (Y or W) chromosome, after recombination of this chromosome has been suppressed, (ii) Single nucleotide polymorphism (SNP)-based methods to identify allelic sex differences, and (iii) a reference-free k-mer analysis to identify sex-specific sequences independently of a reference genome. This integrated approach increases the robustness of sex-linked signal detection, particularly in non-model taxa such as *Phoxinus*.

## Materials and methods

### Collection permits

Specimens were collected in accordance with the relevant federal (LFischVO NRW, BayFiG, AVBayFiG, LFischVO BW) and regional (BezFiVO Schwaben) fisheries regulations as well as federal state and country laws on environmental protection (BNatSchG, LNG NRW). This study did not include experiments on living organisms; therefore, no further permissions from federal animal welfare agencies or ethics commissions were required. Samples in France were collected following the Nagoya protocol (French certificate of compliance TREL2302365S/689). Fishes were euthanized with tricaine mesylate (MS-222) or clove oil, directly after capture.

### Sample collection, DNA extraction, and sequencing

**Fig. 1:**
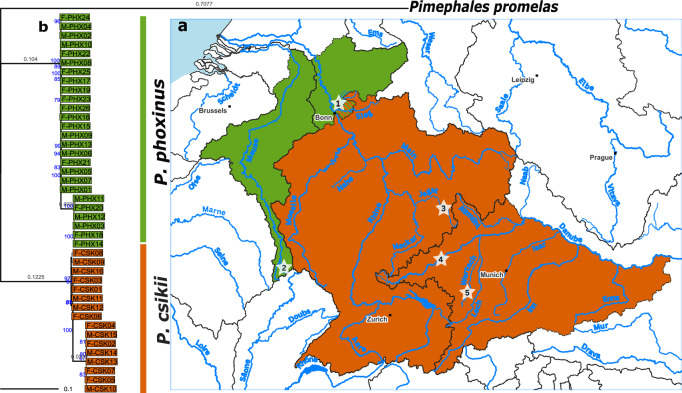
Geographic origin and phylogenetic clustering of analysed samples. **a** Geographic distribution of *P. csikii* (orange) and *P. phoxinus* (green). Sampling drainages are numbered as follows: (1) Middle-Lower Rhine boundary, (2) Meuse, (3) Neckar, 4, (5) Danube. Specific location details can be found in Supplementary Table S1. The to-date known hybrid contact zone is indicated by diagonal stripes. **b** Maximum likelihood phylogeny of *P. csikii* (orange) and *P. phoxinus* (green) based on 12 concatenated mitochondrial protein-coding gene sequences, with Pimephales promelas as an outgroup. Branch lengths are log-transformed. Bootstrap support values ≥ 70% are shown in blue at key nodes, and the longest 5% of branches are labelled with their log-transformed lengths in grey.

Specimens of the two *Phoxinus* species were collected in natural populations in their known range (Sternberg et al. 2025): at the Middle-Lower Rhine boundary and the Meuse drainage for *P. phoxinus* and the Neckar (draining into the Upper Rhine) and Danube drainage for *P. csikii* (Fig. 1a, Supplementary Table S1). Twenty *Phoxinus* at the Middle-Lower Rhine (Agger, Naafbach, and Sülz) were caught with a battery-powered DC electrofishing device (EFGI 650, Bretschneider Special Electronics, Prüm, Germany). Fish from all other sampled drainages were caught using bottletraps, angling and/or dip nets: six *P. phoxinus* specimens from the Meuse (Anger and Mouzon), two *P. csikii* from the Neckar (Jagst draining into the Neckar), and fourteen from the Danube (Wertach and Danube) (geographic details in Fig. 1a and Supplementary Table 1). Fin clips were taken and stored in molecular-grade ethanol (96.6%). In total, 13 males and 13 females of *P. phoxinus* and 8 males and 8 females of *P. csikii* were subjected to whole genome sequencing, which offers the most complete resolution for detecting sex-associated genomic differentiation (Palmer et al. 2019). DNA was extracted using either the single-column based Qiagen DNeasy ® Blood and Tissue Kit or the Qiagen MagAttract® HMW DNA Kit following the manufacturer’s instructions for tissues, lysing the tissues for three hours, and including an RNAse step after lysis. PCR-free libraries were prepared by Novogene GmbH and Macrogen Europe and sequenced on a Novaseq X Plus (PE150) with a targeted 7 to 15 Gb output per sample (details are given in Supplementary Table 1).

### Raw data quality control

Raw sequence reads were assessed for quality using FastQC v0.11.9 (Andrews 2010). Adapter trimming and removal of low-quality reads were then performed with Fastp v0.20.0 (Chen et al. 2018). The trimming parameters included a minimum read length of 25 bp; removal of bases from both the 5′ and 3′ ends with a sliding window of size 1 and a mean quality threshold of 20; fixed removal of 5 bases from both the 5′ and 3′ ends of each read due to biased nucleotide composition in these regions; and a base quality cut-off of 20 for qualified bases.

### Mitogenome assembly and phylogenetic analysis

We assembled and annotated mitochondrial genomes using MitoFinder v1.4.2 (Allio et al. 2020) and the mitochondrial reference genome of *P. phoxinus*, NCBI accession number: *NC_020358* (Imoto et al. 2013) as reference sequence. The *Pimephales promelas* mitogenome was assembled from WGS reads (SRR18560773) for use as an outgroup. Phylogenetic analyses were conducted using concatenated protein-coding genes, namely *cytochrome b* (*CYTB*), *NADH dehydrogenase* subunit genes (*ND1*, *ND2*, *ND3*, *ND4*, *ND4L, ND5*, and *ND6*), *cytochrome c oxidase* subunit genes (*COX1*, *COX2*, and *COX3*), *ATP synthase* subunit genes (*ATP6* and *ATP8*).

All protein-coding sequences were aligned gene-wise using MAFFT v7.453 (Katoh and Standley 2013) and concatenated using AMAS v1.0 (Borowiec 2016) to generate PHYLIP alignments and partitions. The final alignment comprised 11,157 nucleotide positions across all sequences, of which 1096 were parsimony-informative and 1,524 were singleton sites, while 8537 sites were fixed. Maximum likelihood trees were inferred using IQTREE v2.2.2.7 (Minh et al. 2020), with ModelFinder (Kalyaanamoorthy et al. 2017) for model selection and 1000 ultrafast bootstraps (Hoang et al. 2018). The tree was rooted post-inference using the *P. promelas* sample as an outgroup. The resulting tree was visualised in ggtree v1.4.11 (Yu et al. 2017).

### Nuclear-genome based analyses

Sex-associated regions were identified using the SexFindR pipeline (https://github.com/phil-grayson/SexFindR.git), which integrates multiple complementary analyses for robust identification of sex chromosomes (Palmer et al. 2019). This pipeline has been validated in other teleosts (Dubin et al. 2025; S. H. Smith et al. 2025). We reimplemented this pipeline in a reproducible Snakemake workflow (Köster and Rahmann 2012) to streamline execution. Full workflow and complete analysis details are archived at 10.5281/zenodo.16389484.

#### Read mapping, variant detection, and filtering

Cleaned fastq files were aligned to the *P. phoxinus* reference genome (GenBank ID: GCA_037504875.1) (Oriowo et al. 2025) using BWA v2.2.1 (Li 2013). We called variants with bcftools v1.19 (Danecek et al. 2021). To generate a high-quality SNP dataset for downstream analyses, raw bcf files were filtered to retain variants matching the following parameters: “QUAL ≥ 30, read depth 10 - 200, no indels, max 10% missing genotypes”.

#### Coverage-based analyses

To identify sex-limited regions in *P. phoxinus* and *P. csikii*, male and female bam files were merged into one file per sex using SAMtools v1.19.2 (Li et al. 2009). Coverage statistics generated with Samtools provided depth thresholds and an adjustment coefficient (AC) for each individual. DifCover v3.0.1 (J. J. Smith et al. 2018) compared normalised coverage between sexes in 1 kb windows, with log2-transformed ratios adjusted by AC.

The resulting values were used as input for DNAcopy v1.80.0 (Olshen et al. 2004) to segment the genome and identify candidate chromosomes/genomic regions with coverage differences between males and females.

#### SNP-based analyses

Sex-associated regions are expected to show fixed differences between the sex chromosomes, but differences may also occur elsewhere in the genome due to reduced recombination or other evolutionary processes. To identify such regions of divergence, we performed four complementary analyses: Intersex fixation index (F_ST_), sex-biased SNP density, nucleotide diversity (π), and genome-wide association studies (GWAS). Intersex F_ST_ was estimated using VCFtools v0.1.16 (Danecek et al. 2011) on all individual SNPs, which were then ranked from highest to lowest to highlight genomic regions with the greatest differentiation between sexes. SNP density was calculated in 10 kb windows with VCFtools, and male-female means were compared following the SexFindR workflow. Significance was assessed with 100,000 random permutations of sex labels, and windows with p-value ≤ 0.05 were ranked by significance and effect size. Nucleotide diversity was also estimated separately for males and females in 10 kb windows.

GWAS for SNP-phenotypic sex associations was performed with GEMMA v0.98.5 (X. Zhou and Stephens 2012) using PLINK v1.90b6.12 (Chang et al. 2015) formatted genotype data, and significant SNPs were ranked by statistical significance. We integrated results by identifying 10 kb windows enriched for the top 5% SNPs across all four analyses (SNP density, π, GWAS, and F_ST_). The sexfindR pipeline considers the top 100 windows that consistently appear in the results of the window-based analyses of genome-wide FST, GWAS, SNP-density and π to be sex-associated regions.

All results were visualised as Manhattan plots using *ggplot2* v3.5.1 (Wilkinson 2011) in R version v4.4.3 (Ihaka and Gentleman 1996). These plots provided a clear representation of the SNP associations across the genome, highlighting regions of interest with significant differences between sexes. To validate the signals detected in the SNP-based analyses, we performed a randomisation test by randomly assigning phenotypic sex to individuals and re-running the entire pipeline, expecting genuine sex-linked signals to disappear, while signals driven by other types of variation would remain.

#### K-mer-based analyses

Combining SNP-based analyses and k-mer-based GWAS improves power to detect sex-associated variants, including those missed by alignment-based SNP-calling (Rahman et al. 2018). K-mer-based analyses are especially powerful when a reference genome is lacking or is too divergent from the focal species (Shi et al. 2025). We conducted a k-mer-based GWAS analysis following the default SexFindR pipeline with minor modifications. For each sample, 31-mers were counted from filtered reads using KMC3 v3.2.4 (Kokot et al. 2017), separating canonical k-mers ( ≥ 2 counts) from non-canonical. These were merged with strand information into a single file per sample.

For each species, k-mers present in at least five samples and canonical in ≥20% were retained. A binary presence/absence matrix was generated and converted to PLINK format, retaining only k-mers with a minor allele frequency (MAF) ≥ 0.05 and a minor allele count (MAC) ≥ 5. Association testing was performed using PLINK with default settings to compute p-values. The most significant k-mers were parsed using the plink_to_abyss_kmers.py script, also included in the kmerGWAS (Voichek and Weigel 2020) part of the pipeline. These k-mers were then assembled into contigs using ABySS v2.3.7 (Simpson et al. 2009).

To obtain an overview of where the assembled contigs could be located in the reference genome, we used *blastn* from the BLAST + SUITE v2.16.0 (Camacho et al. 2009) after converting the *P. phoxinus* reference genome to a BLAST database using the *makeblastdb* command.

#### Validation of sex-associated regions

Candidate sex-associated regions identified in above described analyses were extracted from genome-wide VCF files using VCFtools (*--chr* and *--from-bp/--to-bp)*. Within these regions, we assessed sex-specific differentiation through three approaches: multidimensional scaling (MDS), genotype heatmaps, and haplotype phylogenies.

For the MDS, a pairwise distance matrix was generated with PLINK, and we then performed classical multidimensional scaling on the distance matrices in R and plotted using ggplot2. To examine individual genotypes, genotype matrices were extracted from vcf files using the R package vcfR v1.15.0 (Knaus and Grünwald 2017), followed by generating heatmaps of genotypes using pheatmap v1.0.12 (Kolde 2015).

Haplotype-phased phylogenies were constructed by first removing sites with missing data with BCFtools, then phasing using BEAGLE v22jul22 (Ayres et al. 2012). For each sample, two full-length consensus sequences representing haplotype 1 and haplotype 2 of the entire sex-associated region were generated using *vcf-consensus* with the -*H 1* and -*H 2* flags, incorporating phased SNPs and filling invariant sites from the reference. These sequences were used to construct maximum likelihood phylogenies in IQ-TREE, with ModelFinder used to select the best-fit substitution model, and node support assessed with 1000 ultrafast bootstrap replicates to provide statistical confidence in the tree topology.

#### Functional annotation of candidate regions

All SNP-based analyses used high-quality SNPs filtered for depth, missingness, and minor allele frequency, but not linkage disequilibrium (LD). In a post hoc analysis, LD-based pruning removed approximately 87% of variants in *P. phoxinus* and 92% in *P. csikii*, indicating extensive LD across large genomic regions in both species. To visualise haplotype structure, primary LD blocks were generated and plotted using LDBlockShow v1.40 (S.-S. Dong et al. 2021) for 100 kb regions upstream and downstream of each candidate region. Candidate genes within these haplotype blocks were annotated by BLAST search against the SwissProt protein database (Boeckmann et al. 2003). For more comprehensive functional annotation, we defined candidate regions as 500 kb windows centred on each significant SNP to maximise coverage of potentially linked genes and variants. While this conservative approach helps prevent missing true candidates, it may also include unrelated genes, reducing the specificity of downstream annotations.

Gene Ontology (GO) and KEGG pathway enrichment analyses of protein-coding genes within these ± 500 kb candidate regions were performed using clusterProfiler v4.10.1 (Yu et al. 2012). Biological processes and KEGG pathway enrichments were tested using Benjamini-Hochberg adjusted *p* < 0.05, and significant terms were visualised with ggplot2.

## Results

### Mitogenome phylogeny of *P. phoxinus* and *P.csikii* specimens

A total of 43 individuals (26 *P. phoxinus*, 16 *P. csikii*, and one *Pimephales promelas* as outgroup) were included in this phylogenetic analysis based on twelve concatenated protein-coding mitochondrial genes. The maximum-likelihood phylogeny showed strong bootstrap support at internal nodes defining both species clusters, demonstrating the monophyly of *P. phoxinus* and *P. csikii* (Fig. 1b). These two distinct clusters confirmed distinct species status and justified subsequent species-specific analyses.

### Sex determination in *P. phoxinus*—evidence for an XY system

#### Read mapping and coverage analysis

A total of 13 male and 13 *P. phoxinus* female specimens yielded 101 million and 88.5 million filtered reads, respectively. After alignment to the reference genome, we obtained mean coverages of 12.7× for males and 11.3× for females. Mapping rates were high and comparable across groups. On average 98.59% of reads aligned to the 25 chromosomes of the reference genome. The coverage-based analysis did not reveal any differentiated sex chromosomes between males and females (Supplementary Fig. S1). Sex-specific coverage variation per chromosome ranged from 0 to 6.11%. Chromosomes 15 and 3 exhibited the highest proportion of coverage variation between the sexes, yet did not reach an expected 50% reduction in one sex if sex chromosomes in the heterogametic sex were differentiated to a point where Y/W reads would not map to X/Z regions (Supplementary Table S2).

#### SNP-based detection of candidate sex-associated regions

**Fig. 2:**
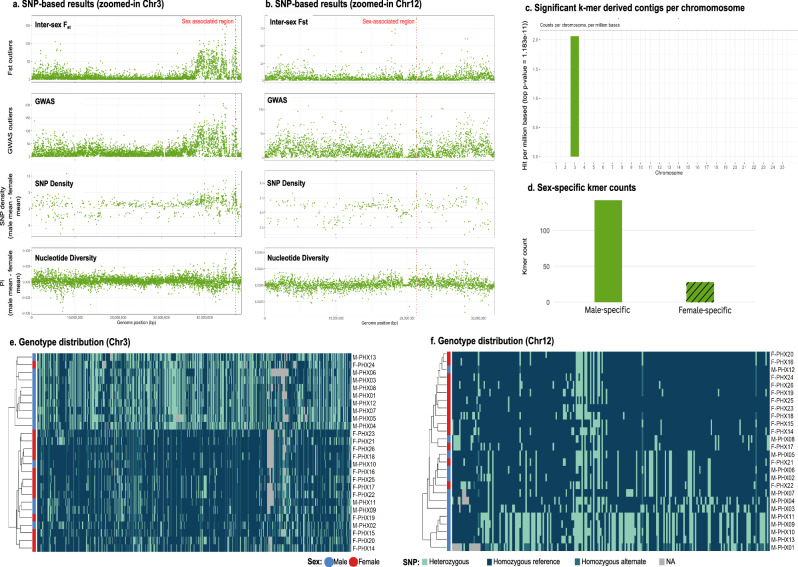
Genomic signals of sex differentiation in *P. phoxinus.* Combined visualisation of SNP-based analysis of sex differentiation. Identified sex-associated windows on (**a**) chromosome 3 (47.14–47.16 Mb) and **b** chromosome 12 (21.27–21.28 Mb) are highlighted with a red line. **c** Results of k-mer-based analysis. Normalised counts of the most significant top k-mers (*P* = 1.18 × 10⁻¹¹) per megabase across all chromosomes. 170 top k-mers were assembled into two contigs. **d** Total counts of significant sex-specific k-mers across the genome. Genotype heatmap for the SNPs within the sex-associated windows identified on (**e**) chromosomes 3 (total of 632 SNPs) and **f** chromosome 12 (total of 225 SNPs). Heterozygous states are shown in turquoise, homozygous reference states in dark blue, and homozygous alternative states in ocean blue. Males show more heterozygosity (turquoise); females are mostly homozygous (dark shades), consistent with an XY sex-determining system.

Variant calling in *P. phoxinus* yielded 38,501,352 SNPs and 5,414,649 indels, and after filtering, ~27 million high-confidence SNPs were retained. The top 5% of SNPs from all analyses were binned into 91,973 non-overlapping 10 kb windows. We identified three candidate sex-associated 10 kb windows: two adjacent windows on chromosome 3 (Chr3: 47,140,000–47,150,000 and 47,150,000–47,160,000), which were merged into a single 20 kb region (47.14–47.16 Mb) for simplicity and contained 632 SNPs (Fig. 2a), and one on chromosome 12 (Chr12: 21,270,000–21,280,000) containing 225 SNPs (Fig. 2b, Supplementary Table S3, Supplementary Fig. 2). SNP density in these candidate windows was male-biased, consistent with an XY system.

The sex randomisation analysis confirmed that the three previously identified sex-linked signals are true sex-linked regions (Supplementary Fig. S3).

#### K-mer-based analysis supports chromosome 3 as a potential sex chromosome

In *P. phoxinus*, the 170 k-mers had the most significant p-values (lowest p-value, 1.183 × 10⁻¹¹). Of these, 142 were male-specific, and 28 were female-specific (Fig. 2d). All 170 k-mers were then assembled into two contigs, both of which mapped to chromosome 3 (positions 46,565,966–46,566,020 bp; Fig. 2c), located within the *gpsm1* gene, a G-protein modulator gene previously associated with spermatogenesis (Avellar et al. 2009; Dai et al. 2024). Among these unassembled k-mers, 142 were male-specific, and 28 were female-specific (Fig. 2d), consistent with the male-biased sequence divergence observed in our SNP-based approaches. Since the initial analysis of sex-associated kmers resulted in only two contigs, we expanded to the top 1% most significant k-mers, which yielded 221,385 k-mers that assembled into 120,402 contigs, allowing us to test for additional genomic regions with potential sex-differential signals. This more extensive k-mer set also exhibited an enrichment on chromosome 3, where 67,023 contigs (55.67%) mapped within the interval of 37,001,252 to 48,464,155 bp, indicating this region as a potential hotspot for sex-associated differentiation in *P. phoxinus* (Supplementary Fig. S4, Supplementary Fig. S5). It is noteworthy that the 20 kb region identified through the SNP-based analyses falls within the same interval, indicating concordance between SNP and k-mer signals.

#### Sex-associated genetic structure in identified candidate regions

Multidimensional scaling (MDS) of genome-wide SNPs revealed clustering primarily by river drainage (Supplementary Fig. S6), reflecting population structure across the sampled regions. In contrast, MDS focused on SNPs within the sex-associated regions on chromosomes 3 and 12 (Chr3: 47.14–47.16 Mb; Chr12: 21.27–21.28 Mb) allowed us to specifically examine variation related to sex. On chromosome 3, Rhine samples showed strong sex-specific clustering, with 18 of 20 individuals correctly grouped by phenotypic sex (10/11 females, 8/9 males). In contrast, Meuse samples showed weaker differentiation, with only 3 of 6 individuals clustering by sex (2/2 females, 1/4 males). The pattern was reversed on chromosome 12, where 13 of 20 Rhine samples were correctly assigned (11/11 females, 2/9 males), while Meuse samples showed stronger sex-association, with 5 of 6 individuals clustering by their phenotypic sex (3/4 males, 2/2 females) (see individuals marked with asterisks in Supplementary Figs. S7 and S8). Together, these results suggest that sex-associated differentiation on chromosome 3 is largely driven by the Rhine population, whereas the signal on chromosome 12 is stronger in the Meuse population.

Haplotype-phased phylogenies of consensus sequences in the 20 kb sex associated region on chromosome 3 and the 10 kb region on chromosome 12 showed that male individuals exhibit greater divergence between their two haplotypes, while female haplotypes demonstrated homogeneity (Supplementary Fig. S9, Supplementary Fig. S10).

Analysis of genotypes in the sex-associated region on chromosomes 3 and 12 revealed drainage-specific enrichment of sex-association patterns, supporting the presence of population-specific sex-associated loci in *P. phoxinus*. On chromosome 3, 210 out of 632 SNPs showed ≥50% heterozygosity in males and ≥50% homozygosity in females across all samples (Fig. 2e). This pattern was strongly concentrated in the Rhine drainage, where 227 SNPs met this criterion. In contrast, on chromosome 12, only 10 SNPs showed this pattern overall, yet within the Meuse drainage, 72 SNPs met the ≥50% male heterozygosity and ≥50% female homozygosity threshold (Fig. 2f). These findings indicate that different genomic regions may be involved in sex determination in different drainages, consistent with the presence of population-specific sex-determining systems or recent turnover events.

These findings reveal population-specific sex-association differentiation in *P. phoxinus*, with chromosome 3 showing strong XY patterns primarily in the Rhine drainage, and chromosome 12 showing similar XY patterns mostly in the Meuse drainage.

#### Functional annotation of candidate genes

LD block analysis on chromosome 3 sex-associated region revealed a strongly linked 100 kb region that contained just four genes (*rab14*, *abl1*, *exosc2*, and *spr*) (Supplementary Fig. S11). Notably, the core of this region is a gene desert, indicating limited coding content within the primary haploblock. To comprehensively assess potential functional elements, we expanded our annotation window to 500 kb centred on the region of interest (Supplementary Table S4). Despite the core interval’s limited gene content, gene ontology (GO) enrichment analysis of the expanded 500 kb window identified 29 significantly enriched terms, including “regulation of Ras protein signal transduction,” “regulation of animal organ morphogenesis,” and “positive regulation of Ras protein signal transduction” (Supplementary Fig. S12, Supplementary Table S5).

On chromosome 12, LD analysis identified a strongly linked 100 kb window containing seven genes: *gabrr2*, *rragd*, *syne*, *map7*, *clmn*, *gja10*, and *bach2* (Supplementary Fig. S13). To ensure broad functional annotation, we again expanded the candidate region to 500 kb; full gene lists are provided (Supplementary Table S6) and Gene Ontology (GO) enrichment analysis of the expanded 500 kb candidate region returned terms prominently related to TOR (Target of Rapamycin) signalling, including “TOR signalling,” “regulation of TOR signalling,” “positive regulation of TORC1 signalling,” and “positive regulation of TOR signalling” (Supplementary Fig. S14, Supplementary Table S7).

### Sex determination in *P. csikii*—evidence for a ZW system

#### Read mapping and coverage analysis

A total of 89.1 million and 78.5 million filtered reads were generated from 8 male and 8 female *P. csikii* individuals, respectively. The mapping rate was found to be 98.42% and 98.35% for the male and female samples, respectively, with a mean coverage of 15.81-fold and 17.94-fold in males and females, respectively. The coverage analysis that identifies chromosome proportions with significantly different coverage between male and female samples yielded, as in *P. phoxinus*, no conclusive results. The range of sex-specific coverage variation per chromosome ranged from 0% to 2.52% (Supplementary Table S8). Chromosomes 3 and 15 exhibited the highest proportion of coverage variation between the sexes (Supplementary Fig. S15).

#### SNP-based analyses find no candidate sex-associated regions

From a total of 29,677,630 SNPs and 4,691,776 indels, ~18 million high-confidence variants were retained after quality filtering and the top 5% of SNPs were binned into 91,808 non-overlapping 10 kb windows. These went into our SNP-based analyses for detecting genomic sex-associated differentiation. As with *P. phoxinus*, we independently ranked the SNPs for each analysis, but none of the ranked 10 kb windows appeared as an outlier across all four SNP-based metrics (F_ST_, GWAS, π, SNP density, Supplementary Fig. S16). Hence no candidate sex-associated region was identified.

To ensure that the non-significant signal detected in the SNP-based analyses (Supplementary Fig. S16, Chr22) is not related to sex determination, we further conducted a randomised sex-reassignment analysis. The results underlined that the weak signal is not associated with sex as it persisted (Supplementary Fig. S17).

#### K-mer-based analysis identifies chromosome 3 as potential sex chromosome

**Fig. 3:**
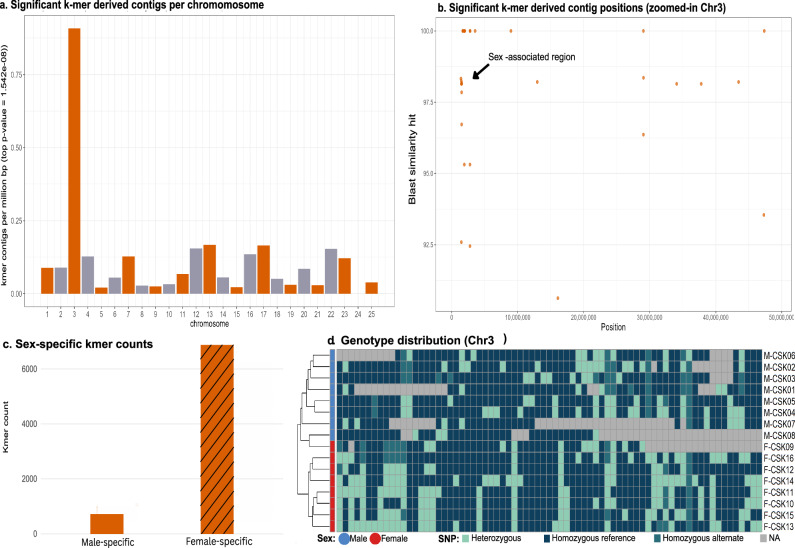
Genomic signals of sex differentiation in *P. csikii.* **a** Results of k-mer-based analysis. Normalised counts of the most significant top k-mers (*P* = 1.54 × 10⁻⁸) per megabase across all chromosomes. 7583 top k-mers were assembled into 133 contigs across all chromosomes but chromosome 24. **b** Genomic distribution of these k-mer contigs along chromosome 3a and the highlighted sex-associated region. **c** Total counts of significant sex-specific k-mers across the genome. **d** Genotype heatmap for the 76 SNPs within the sex-associated region identified on chromosome 3 (from 1.493 to 1.494 Mb). Darker boxes indicate homozygosity (reference states in dark blue and homozygous alternative states in ocean blue), lighter shades (turquoise) indicate heterozygosity. Males are mostly homozygous, while females show more heterozygous genotypes, consistent with a ZW system.

In *P. csikii*, the most significant (top) sex-associated k-mers had a *p* value of 1.542 × 10⁻⁸. A total of 7583 k-mers were assembled into 133 contigs, which mapped to multiple chromosomes in the *P. phoxinus* reference genome, with a notable enrichment on chromosome 3 (Fig. 3a). Two regions on chromosome 3 (1,492,000 to 1,499,081 bp and 1,950,070 to 1,953,680 bp) showed the highest density of mapped contigs (Fig. 3b). Of the 133 contigs, 63 (47.4%) had at least one BLAST hit to the reference genome. In contrast to *P. phoxinus*, the k-mer-based analysis of the most significant k-mers in *P. csikii* yielded already sufficient signal, so we did not expand the analysis to the top 1% k-mers. Of the 7583 significant k-mers, 6864 (90.52%) were female-specific and 719 (9.48%) were male-specific (Fig. 3c).

#### Sex-associated genetic structure in candidate regions

Multidimensional scaling (MDS) of ~18 million genome-wide SNPs shows clustering by river drainage, reflecting population structure across the sampled regions (Supplementary Fig. S18). Of the two k-mer-enriched regions on chromosome 3, the latter (Chr3:1,950,070 to 1,953,680) contained no SNPs. Within the first region (Chr3:1,490,000 to 1,499,081), though, we found 76 SNPs that accumulated in a narrow ~ 1 kb region from 1,492,997 to 1,493,793 bp, so we focused on this interval in subsequent analyses. MDS plots of those 76 SNPs showed clear separation by phenotypic sex (8/8 females, 8/8 males) (Supplementary Fig. S19). A haplotype-phased phylogeny showed divergence between both female haplotypes, while male haplotypes collapsed (Supplementary Fig. S20). 21 of the 76 SNPs had ≥50% heterozygosity in females and ≥50% homozygosity in males. Visual inspection of SNP genotypes in this candidate region shows exactly this: predominant heterozygosity in females, whereas homozygosity was prevalent among male samples (Fig. 3d).

#### Functional annotation of candidate genes

LD block analysis on *P. csikii* chromosome 3 detected a 250 kb haploblock spanning the sex-associated region (Supplementary Fig. S21). Within this interval, we identified 14 candidate genes (*cit, rab35, gng10, rabepk, hspa5, fastkd5, ddrgk1, haus4, fubp3, ass1, surf4, surf2, bbln*, and *ciz1*). To ensure comprehensive characterisation of potentially linked elements, we expanded our annotation window to 500 kb on either side of the association peak, which included 23 genes in total (Supplementary Table S9). Gene Ontology (GO) enrichment analysis within this expanded region identified significant terms related to lipid metabolism and stress response, including “phospholipid metabolic process,” “glycerophospholipid metabolic process,” and “regulation of IRE1-mediated unfolded protein response” (Supplementary Fig. S22, Table S10). KEGG pathway analysis, in contrast, found enrichment specifically for “ovarian steroidogenesis” and “GnRH signalling pathway” (Supplementary Fig. S23, Table S11).

## Discussion

We investigated the genotypic basis of sex determination in two closely related species, *P. phoxinus* and *P. csikii*, using 26 and 16 whole genomes, respectively. To identify sex-associated regions, we applied complementary analyses based on coverage, SNPs, and k-mers. Coverage analyses did not reveal any chromosomes with significant sex-associated differences in either species, and these regions did not overlap with sex-associated regions identified by SNP or k-mer analyses, consistent with karyotypic studies reporting no visibly differentiated sex chromosomes in *Phoxinus* (Boroń 2001; Joswiak et al. 1980).

In *P. phoxinus*, both SNP- and k-mer-based approaches pointed to sex-associated regions on chromosome 3 and chromosome 12. For *P. csikii*, only the reference-free k-mer analysis identified two sex-associated regions on chromosome 3. These sex-associated regions are not syntenic nor homologous between the species: the 20 kb region in *P. phoxinus* is located near the end of chromosome 3, whereas both corresponding regions in *P. csikii* are near the start. Most of the second *P. csikii* sex-associated region (1,950,070–1,953,680 bp) lacked SNP calls when mapped to the *P. phoxinus* reference genome.

K-mer analysis revealed more male-specific sequences in *P. phoxinus*, whereas *P. csikii* harboured a higher proportion of female-specific k-mer sequences, consistent with the contrasting XX/XY and ZZ/ZW revealed by the haplotype structure: *P. phoxinus* shows signatures of male heterogamety (XX/XY) with clear X–Y haplotype divergence, while *P. csikii* displays evidence of female heterogamety (ZZ/ZW) with distinct Z–W divergence. The sex-associated regions also differ in gene content; despite these differences, gene ontology analyses suggest that conserved gene functions underlie sex determination in both species.

### Evidence for male heterogamety in *P. phoxinus*

In *P. phoxinus*, male-heterozygous SNPs overlap with male-specific k-mers, strongly supporting an XX/XY system. Multiple matrices, including F_ST_, GWAS, nucleotide diversity, and SNP density, corroborated these signals. A region on chromosome 3 correctly predicted phenotypic sex for 21 of 26 individuals, primarily from Rhine populations, while a second region on chromosome 12 correctly assigned the remaining five individuals, which mostly (3 out of 5) originated from the Meuse. *P. Phoxinus* showed geographically restricted, and hence likely population-specific, sex-associated loci. This mirrors a well-documented feature of teleost fishes, where sex-linked regions often vary or undergo rapid turnover across lineages (Ferreira et al. 2016). We note that sex-association across both regions exhibits incomplete penetrance at the population level, with occasional genotype-phenotype mismatches observed. Such mismatches have been documented in *Culaea inconstans* populations (Pigott et al. 2025). However, we consider sex reversal to be an unlikely explanation given that *P. phoxinus* is a gonochoristic species (Rasotto et al. 1987).

Genes in the the sex-associated regions on chromosomes 3 and 12 in *P. phoxinus* did not match known master sex-determining genes. The sex-associated region in chromosome 3 contained notable candidates such as (i) *rab14*, a Ras superfamily member that regulates cellular trafficking and cell growth and is linked to spermatogenesis and sperm motility in mammals (Bae et al. 2019, 2022); (ii) *abl1*, required for sperm capacitation and maintenance of germ cell genomic integrity (Baker et al. 2009); and (iii) *exosc2*, a core component of the RNA exosome complex that regulates RNA turnover and cell fates (Srinivasan et al. 2024). Mutations in *exosc2* can disrupt RNA metabolism, altering the expression of developmental genes and potentially sex differentiation genes, such as *sox5* (Yang et al. 2020). This region also contains (iv) *spr* (sepiapterin reductase), a key enzyme in the pteridine pathway responsible for producing yellow-orange pigmentation (Tian et al. 2024). Pigmentation genes often occur in sex-linked regions and are tied to sex-specific traits or antagonistic selection (Kottler and Schartl 2018). In *P. phoxinus*, sexual dichromatism is subtle, though males show seasonal nuptial coloration (intensified red belly and fin pigmentation with enhanced golden flanks) (Frost 1943), suggesting sex-specific regulation. While *spr*’s presence does not prove a role in sex determination, its repeated association with sex-linked regions in teleosts, including guppies (Künstner et al. 2016), supports the idea that pigmentation genes may be linked to sex chromosomes through indirect selection on sex-specific traits.

On chromosome 12, several genes are linked to cellular regulation and fertility. (i) *gabrr2* encodes a G-protein-coupled receptor important for cell proliferation and maintaining spermatogonial stem cells (Bettler et al. 2004). It can act as a negative regulator of cell proliferation in these cells (Du et al. 2013), and may also transduce environmental signals that stimulate gonadal development (Ma et al. 2023). (ii) *rragd* encodes a GTP-binding protein that mediates amino acid-responsive mTORC1 signalling, a pathway essential for cellular growth and metabolism (Tsun et al. 2013). Lastly, (iii) *syne3*, a component of the LINC complex, is vital for the structural linkage between the sperm head and tail, a process crucial for proper sperm formation and male fertility (Zhang et al. 2021).

Together, both sex-linked regions harbour genes central to germ cell development, sperm function, and cellular growth regulation, suggesting that despite their genomic separation, they converge on pathways critical for male fertility and sex determination in *P. phoxinus*.

### Evidence for female heterogamety in *P. csikii*

SNP-based analyses did not identify any 10 kb region significantly associated with phenotypic sex in *P. csikii*. This likely reflects the combined effects of using a *P. phoxinus* reference genome, which may introduce mapping bias and SNP calling bias (Thorburn et al. 2023, Maurstad et al. 2025) and the relatively small *P. csikii* sample size, both of which reduce the power to detect sex-associated loci. However, sample size may be less limiting, as studies have shown that three to eight individuals per sex can suffice to detect sex-linked signals in species with genotypic sex determination (Kirkpatrick et al. 2022). Since the reference genome was generated from a male individual, W-linked sequences unique to females may be absent from the assembly, and thus female-specific reads from such regions would either fail to map or align poorly. This limitation is not unique to our study but is a known challenge in sex determination research (Pyne et al. 2025). Another possibility is that sex, in *P. csikii*, is not determined by genetic factors to the degree it is in *P. phoxinus*, but that environmental factors might additionally play a strong role. This seems unlikely, however, given the 100% differentiation of phenotypic sexes by the identified sex-associated region.

An overrepresentation of female-linked k-mers indicates sequences that are either absent or highly diverged in males. In *P. csikii*, several of contigs derived from sex-specific kmers overlapped regions harbouring female-heterozygous SNPs, providing converging evidence supporting a ZZ/ZW sex determination system. In general, k-mer-based analyses are a powerful tool to reveal sex-associated loci independently of a reference genome, and have been successfully used to identify ZW systems and track rapid sex chromosome turnover in other taxa, including East African cichlids (Behrens et al. 2022; S. H. Smith et al. 2025). The contrast between the lack of a clear SNP-based signal and the presence of strong k-mer signals underscores the limitations of reference-based methods and highlights the effectiveness of alignment-free approaches in detecting sex-associated variation in non-model systems.

Within the female-linked region on chromosome 3, we identified several genes associated with spermatogenesis. While this may seem counterintuitive, such patterns are expected in young sex chromosomes, which often retain ancestrally male-biased expression due to incomplete silencing during early stages of sex chromosome evolution (Larson et al. 2018). (i) *hspa5*, a heat shock protein, regulates protein folding and ER stress responses and is essential for spermatogenesis and male fertility (Nelson et al. 2022). (ii) *ass1*, an enzyme of the urea cycle, converts aspartate, citrulline, and ATP into arginosuccinate, detoxifying ammonia and contributing to arginine biosynthesis (Haines et al. 2010), however, to the best of our knowledge it has no established roles in sex determination. (iii) *rab35* and (iv) *rabbek*, members of the Rab GTPase family, regulate intracellular trafficking, endosomal recycling, and cytoskeletal organization and play key roles in sperm function as well as in male and female meiosis (Bae et al. 2019, 2022; Shan et al. 2021). These candidates suggest potential sexually antagonistic selection, hitchhiking effects or male-biased roles in germ cell function and development, all pending functional validation.

Gene ontology enrichment pointed to significant involvement of “phospholipid metabolic processes”, a pathway known to influence gonad development and steroid hormone biosynthesis (Ye et al. 2021). Studies in *Sebastes melanops* and *Onychostoma macrolepis* show that changes in phospholipid pathways and dietary lipid levels influence gonad development (J. Zhou et al. 2022). KEGG pathway analysis further supported associations with “ovarian steroidogenesis” and “GnRH signalling”, two critical hormonal axes involved in oocyte maturation and female reproductive function in fish (Z. Dong et al. 2022).

### Comparative perspective and evolutionary implications

The identification of distinct sex chromosome systems in *P. phoxinus* and *P. csikii* provides insight into the evolutionary plasticity of sex chromosomes, even among closely related taxa. While *P. phoxinus* exhibits patterns consistent with an XX/XY system, *P. csikii* shows evidence of a ZZ/ZW system, with female-specific k-mers and W-linked sequence signatures. These findings underscore the notion that, even in the presence of relatively shallow divergence and overlapping distribution ranges, teleost fish species can evolve different genetic mechanisms of sex determination. This pattern mirrors observations in other taxa. For example, the *Oreochromis niloticus* (Nile tilapia) possesses the XX/XY system (Taslima et al. 2021), while a ZZ/ZW system is observed in *Oreochromis aureus* (Wu et al. 2021). Closely related members within the genus *Hippoglossus* also exhibit different sex determination systems (Ferchaud et al. 2022). Notably, despite these structural differences in *P. csikii* and *P. phoxinus*, we observed convergence at the level of gene function: both species showed enrichment of genes involved in Ras/Rab-family GTPase activity, and G-protein coupled receptor pathways, which are known to regulate steroidogenesis and gonadal development. This suggests that while the sex-determining architecture may diverge, the downstream developmental pathways may remain partially conserved.

Divergence in sex chromosome systems between closely related species can generate postzygotic reproductive barriers. When taxa with different sex determination systems, such as XX/XY versus ZZ/ZW, hybridize, their offspring inherit novel combinations of sex chromosomes (e.g., XZ, XW, YZ, or YW). These combinations can lead to improper chromosome pairing during meiosis, dosage imbalances, reduced hybrid fitness, altered sex ratios, or sterility and unviability (Ravinet et al. 2017; Sember et al. 2021). Disruption of co-adapted nuclear–mitochondrial interactions may further compromise hybrid viability. Similar patterns are documented in other teleost fish taxa. For example, in *Gambusia holbrooki* (XY) × *Gambusia affinis* (ZW) crosses, the dominant *G. holbrooki* Y chromosome overrides the W, producing male-biased WY hybrids and potentially inviable WW offspring (Kottler et al. 2020). In crosses involving cichlid *Pundamilia pundamilia* and *P. nyererei* females with *Neochromis omnicaeruleus* males, F2 offspring of a rare F1 selfing hermaphrodite exhibited high mortality and female-biased sex ratios (Svensson et al. 2016). These findings underscore that hybridization outcomes are highly context-dependent, shaped by the interactions among X, Y, Z, and W chromosomes and the degree of divergence in sex determination systems.

The presence of two different sex chromosome systems (XY and ZW) in *P. phoxinus* and *P. csikii* suggests that sex chromosome incompatibilities could arise through postzygotic isolation. Hybrids could have genotypes such as XZ, XW, YZ, YW, ZZ, or WW, depending on the cross direction. Studies in teleosts show such combinations can disrupt pairing, dosage balance, or produce unstable sex determination (e.g., *Xiphophorus* hybrids: XX, XW, YW females; XY, YY males). If this were the case in both *Phoxinus* species, we could expect unpredictable hybrid fitness, skewed sex ratios, and skewed mitochondrial inheritance, which together could ultimately act as an important mechanism maintaining species boundaries, even in sympatry or contact zones (Ravinet et al. 2017, 2018). While such a contact zone of both species exists in the Middle Rhine (Sternberg et al. 2025), empirical confirmation of hybridization is currently limited to the combined analyses of mitochondrial *cytb* and *coi*, and nuclear *rho* and *rag1* genes (Palandačić et al. 2017). More comprehensive multilocus or genomic investigations into hybridization between these species have not yet been reported.

Overall, our findings suggest that sex chromosome systems can shift quickly and unpredictably, and that the observed variation in sex-determining systems may be involved in maintaining species boundaries or in speciation itself. Although this study does not directly assess hybrid incompatibility, characterizing sex determination systems in each species lays the groundwork for investigating sex-linked barriers to reproductive isolation in future work.

## Conclusions

The present study reveals that *P. phoxinus* and *P. csikii* possess distinct sex determination systems, with evidence for an XX/XY in *P. phoxinus* and a ZZ/ZW system in *P. csikii*. This divergence, despite their close relatedness, highlights the evolutionary flexibility of sex chromosomes in teleosts. Notably, such disparities may contribute to postzygotic barriers in hybrids, thereby reinforcing reproductive isolation and facilitating speciation or maintaining species boundaries. Collectively, these findings offer novel insights into the genomic basis of sex determination in a non-model hybridizing teleost fish lineage.

## Supplementary information

Supplementary information

Supplementary information

## Data Availability

All raw sequencing data generated in this study have been deposited in the NCBI Sequence Read Archive (SRA) under BioProject accession PRJNA1288365. The assembled mitochondrial genomes and their annotations are available in GenBank under accession numbers PV942571 to PV942613. Sample metadata is contained in Supplementary Table 1. All scripts used for data processing, analysis, and visualization are publicly available on Zenodo at 10.5281/zenodo.16389484.
