# Supplementary material for: Different sex determination systems in two closely related Eurasian minnow (Phoxinus) species
Source: Heredity (Edinb). 2026 Feb 24;135(4):259–70. doi: 10.1038/s41437-026-00827-8 (PMC13102953; doi:10.1038/s41437-026-00827-8)
Supplement: Supplementary file 2 — Supplementary information [file 41437_2026_827_MOESM2_ESM.pdf]

Supplemental Information for:

Different Sex Determination Systems in Two Closely Related *Phoxinus* Species

Temitope Opeyemi Oriowo, Sophie Helen Smith, Jana Thorman, Nils Sternberg, Astrid Böhne, Madlen Stange

Leibniz Institute for the Analysis of Biodiversity Change, Museum Koenig Bonn, 53113 Bonn, Germany

Table of Contents:

Supplementary Figures S1-S23 **3**

Supplementary Figure S1: *P. phoxinus* Difcover Result. 3

Supplementary Figure S2: *P. phoxinus* SNP-based Results 4

Supplementary Figure S3: *P. phoxinus* Randomised-sex SNP-based Results 5

Supplementary Figure S4: Counts of top 1% k-mer derived contigs per chromosome identified in *P. phoxinus* 6

Supplementary Figure S5: Distribution of top 1% k-mers derived contigs on chromosome 3 identified in *P. phoxinus* 8

Supplementary Figure S6: *P. phoxinus* MDS (Full SNPs dataset) 9

Supplementary Figure S7: *P. phoxinus* MDS (Chromosome 3) 11

Supplementary Figure S8: *P. phoxinus* MDS (Chromosome 12) 12

Supplementary Figure S9: *P. phoxinus* Haplotype Phylogeny (Chromosome 3) 13

Supplementary Figure S10: *P. phoxinus* Haplotype Phylogeny (Chromosome 12) 14

Supplementary Figure S11: *P. phoxinus* Linkage Block (Chromosome 3) 15

Supplementary Figure S12: *P. phoxinus* Gene Ontology (Chromosome 3) 16

Supplementary Figure S13: P. phoxinus Linkage Block (Chromosome 12) 17

Supplementary Figure S14: *P. phoxinus* Gene Ontology (Chromosome 12) 18

Supplementary Figure S15: *P. csikii* Difcover Result. 19

Supplementary Figure S16: *P. csikii* SNP-based Results 20

Supplementary Figure S17: *P. csikii* randomised-sex SNP-based Results 21

Supplementary Figure S18: *P. csikii* MDS (Full SNPs dataset) 23

Supplementary Figure S19: *P. csikii* MDS (Chromosome 3) 24

Supplementary Figure S20: *P. csikii* Haplotype-phased Phylogeny (Chromosome 3)25

Supplementary Figure S21: *P. csikii* Linkage Block (Chr3) 27

Supplementary Figure S22: *P. csikii* Gene Ontology (Chr3) 28

Supplementary Figure S23: *P. csikii* Gene Ontology (Chr 3) 29

Supplementary Tables S2-S10 **30**

Supplementary Table S2: Summary of Sex-associated Coverage by chromosome in *Phoxinus phoxinus* 30

Supplementary Table S3: Genomic Regions Highlighted by SNP Density, F_{ST}, GWAS, and Nucleotide Diversity Rankings in *P. phoxinus* 31

Supplementary Table S4: Genes Located 500 kb Upstream and Downstream of the *P. phoxinus* Candidate Sex-associated region on Chromosome 3 32

Supplementary Table S5: Enriched Gene Ontology (GO) Terms Among Genes Adjacent to the *P. phoxinus* Sex-associated region on chromosome 3 33

Supplementary Table S7: Enriched Gene Ontology (GO) Terms Among Genes Adjacent to the *P. phoxinus* Sex-associated region on chromosome 12 37

Supplementary Table S8: Summary of Sex-associated Coverage by chromosome in *Phoxinus csikii* 38

Supplementary Table S9: Genes Located 500 kb Upstream and Downstream of the *P. csikii* Candidate Sex-associated region on Chromosome 3 40

Supplementary Table S10: Genes Located 500 kb Upstream and Downstream of the *P. csikii* Candidate Sex-associated region on Chromosome 3 41

Supplementary Figures S1-S23

Supplementary Figure S1: *P. phoxinus* Difcover Result.

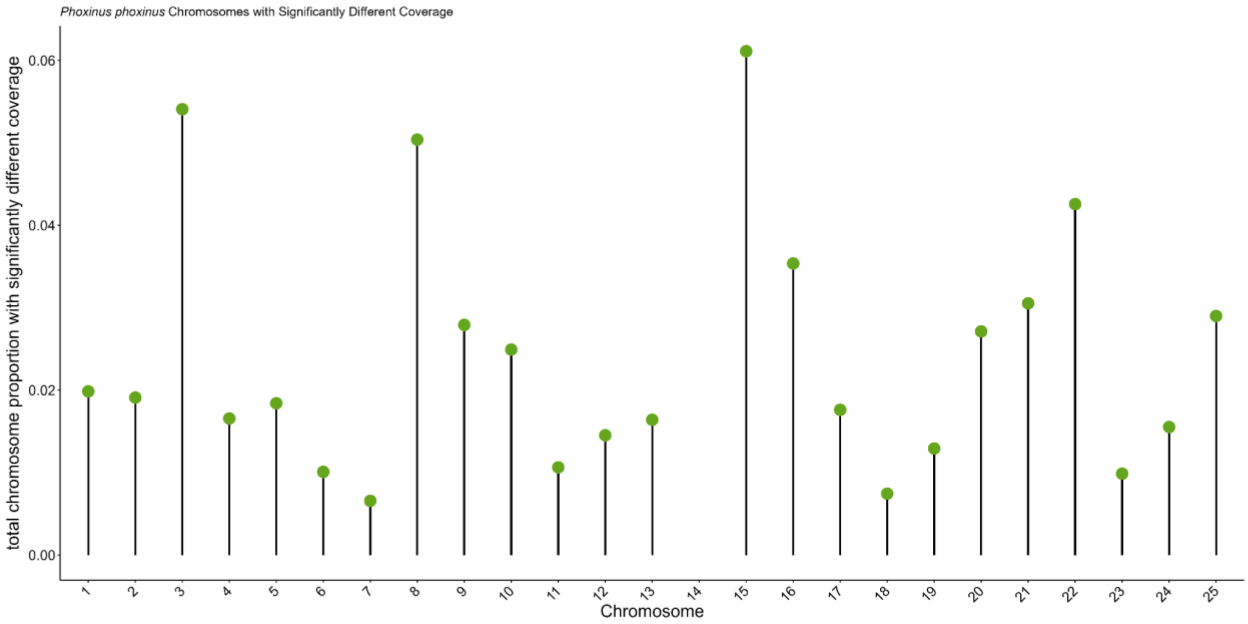

Figure S1. Coverage analysis reveals no highly differentiated sex chromomosomes between males and females in *P. phoxinus*. The plot above highlights chromosome proportions with significantly different coverages between male and female samples in *Phoxinus phoxinus*. Chromosomes 3, 8, and 15 show the most pronounced differences in coverage, albeit small, with 5.3%, 5.0%, and 6.1% of their regions with significant coverage differences between sexes, respectively. No region of chromosome 14 showed substantial differences in coverage between males and females.

Supplementary Figure S2: *P. phoxinus* SNP-based Results

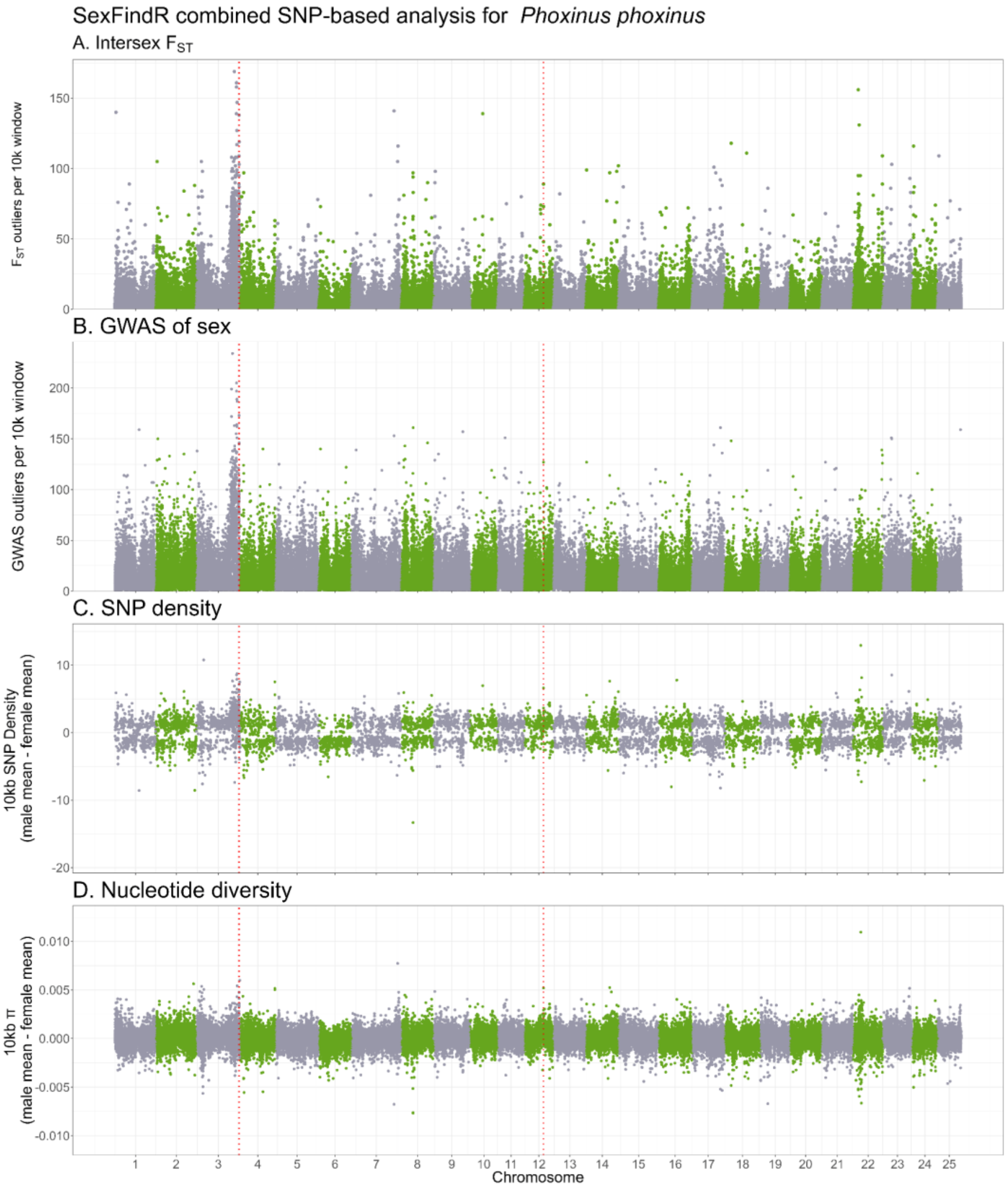

Figure S2. Results from four SNP-based analyses — F_{ST}, GWAS, SNP density, and nucleotide diversity — are arranged from top to bottom, with detected sex-associated regions highlighted. These analyses are based on approximately 28 million SNPs evaluated in 10 kb windows for *P. phoxinus*. Red lines highlight regions where signals jointly derived from all four analyses were detected, identifying sex-associated regions on chromosomes 3 and 12. Outliers for Fst and GWAS are defined as the top 5% of observed values.

Supplementary Figure S3: *P. phoxinus* Randomised-sex SNP-based Results

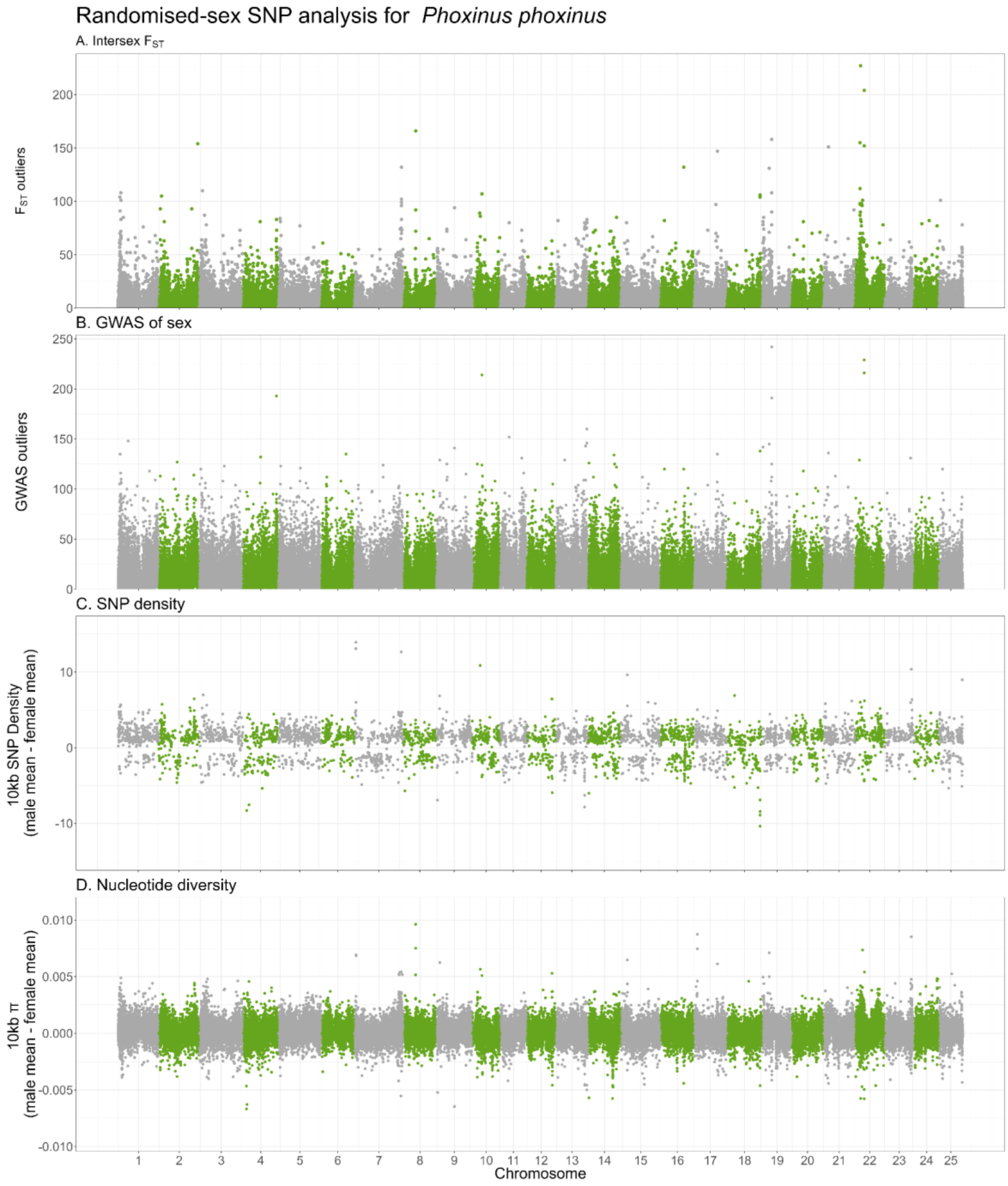

Figure S3. The results from four SNP-based analyses on randomised phenotypic sex labels – FST, GWAS, SNP density, and nucleotide diversity – are arranged from top to bottom. Following the randomisation of sex labels, no sex-associated region from Figure S2 remained, only chromosome 22 retained signals, indicati. These analyses are based on approximately 28 million (SNPs), evaluated in 10-kb windows, for *P. phoxinus*.

Supplementary Figure S4: Counts of top 1% k-mer derived contigs per chromosome identified in *P. phoxinus*

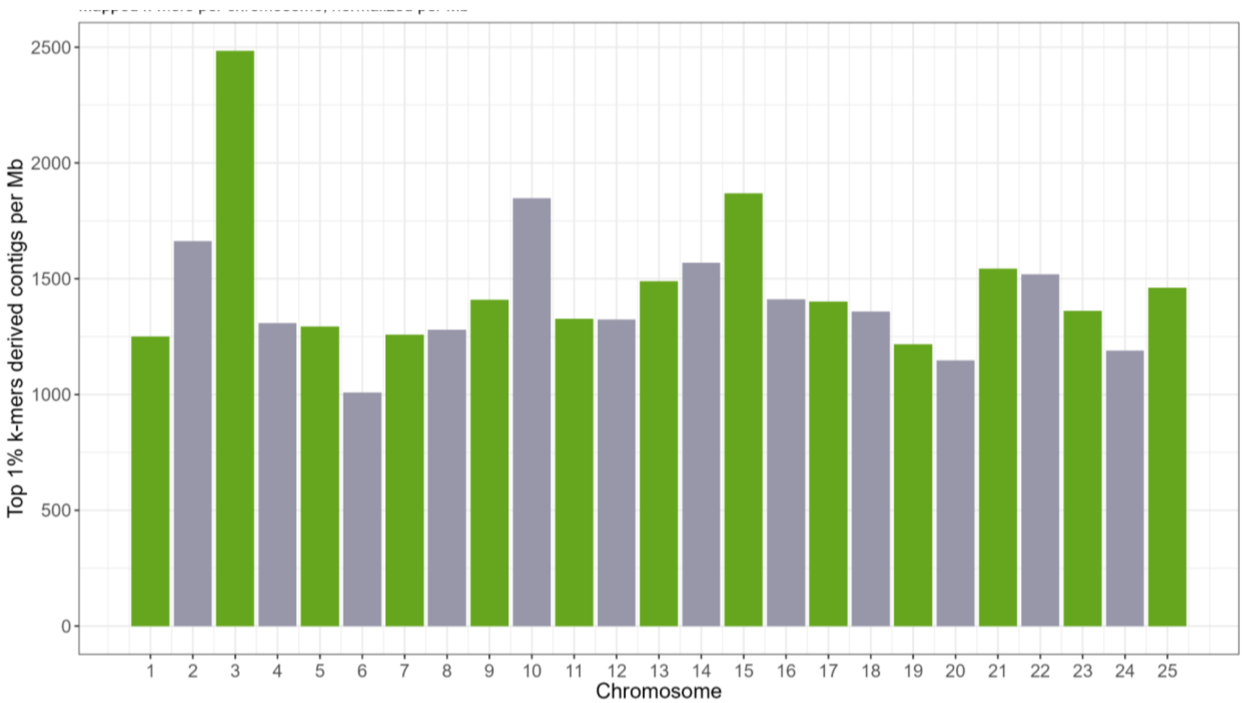

Figure S4: Normalized counts of the top 1% most significant k-mers per million bases across chromosomes in *P. phoxinus*. Chromosome 3 shows a clear overrepresentation of significant sex-linked k-mers.

Supplementary Figure S5: Distribution of top 1% k-mers derived contigs on chromosome 3 identified in *P. phoxinus*

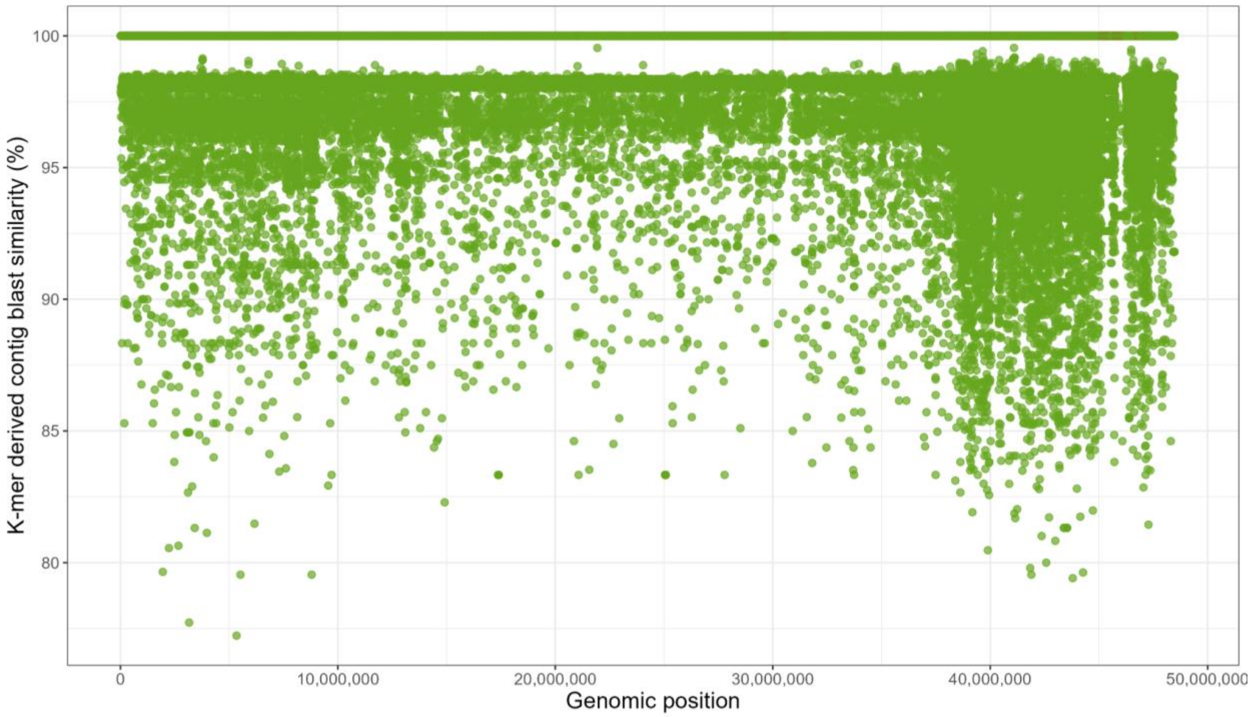

Figure S5: Genomic positions of the top 1% most significant k-mers along chromosome 3 in *P. phoxinus.*

Supplementary Figure S6: *P. phoxinus* MDS (Full SNPs dataset)

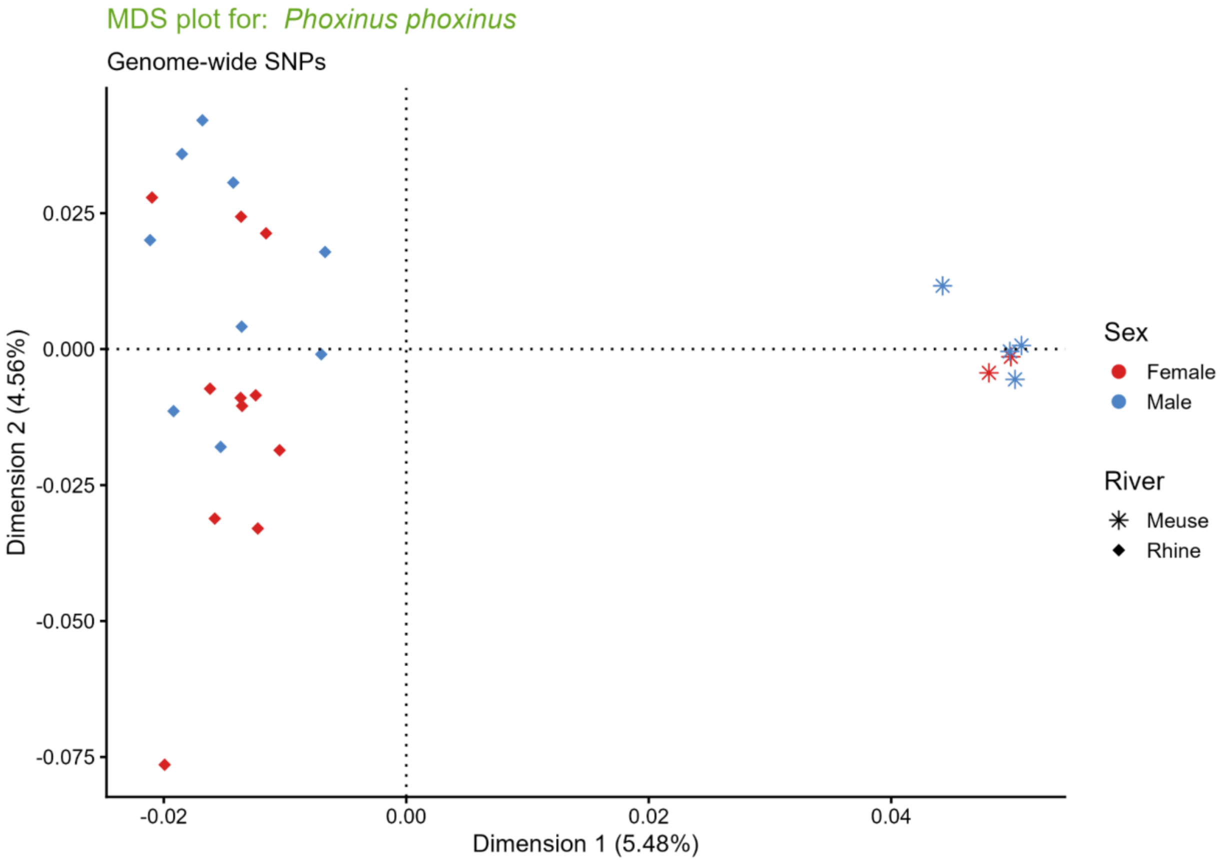

Figure S6. Multidimensional scaling (MDS) of ~28 million genome-wide SNPs in *P. phoxinus* from the Rhine and Meuse drainages. The plot shows the first two MDS dimensions based on pairwise genetic distances calculated from approximately 28 million SNPs. Diamond-shaped points represent Rhine samples, while star-shaped points represent Meuse samples. Individuals are observed to cluster according to the sampled drainage population rather than exhibiting any sex-linked patterns on a genome-wide scale.

Supplementary Figure S7: *P. phoxinus* MDS (Chromosome 3)

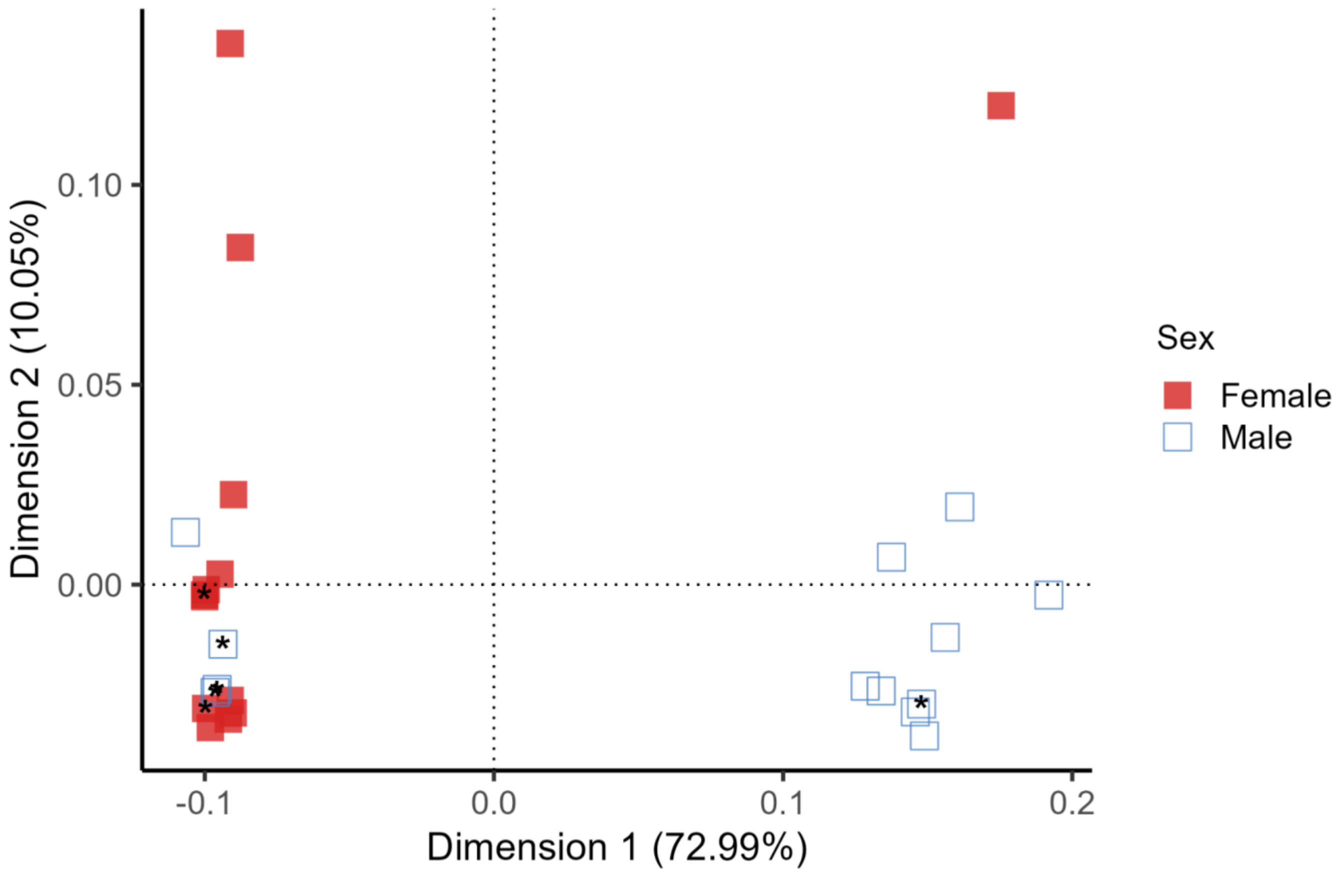

Figure S7. Clustering of 632 SNPs according to phenotypic sex within a sex-associated region (47.14 - 47.16 Mb) on chromosome 3 in *P. phoxinus*. Phenotypic sex was inferred by visual inspection of the gonads. The * symbol indicates *P. phoxinus* samples from the Meuse drainage. Most samples cluster according to expected phenotypic sex within this region, suggesting a strong sex association, particularly in Rhine populations.

Supplementary Figure S8: *P. phoxinus* MDS (Chromosome 12)

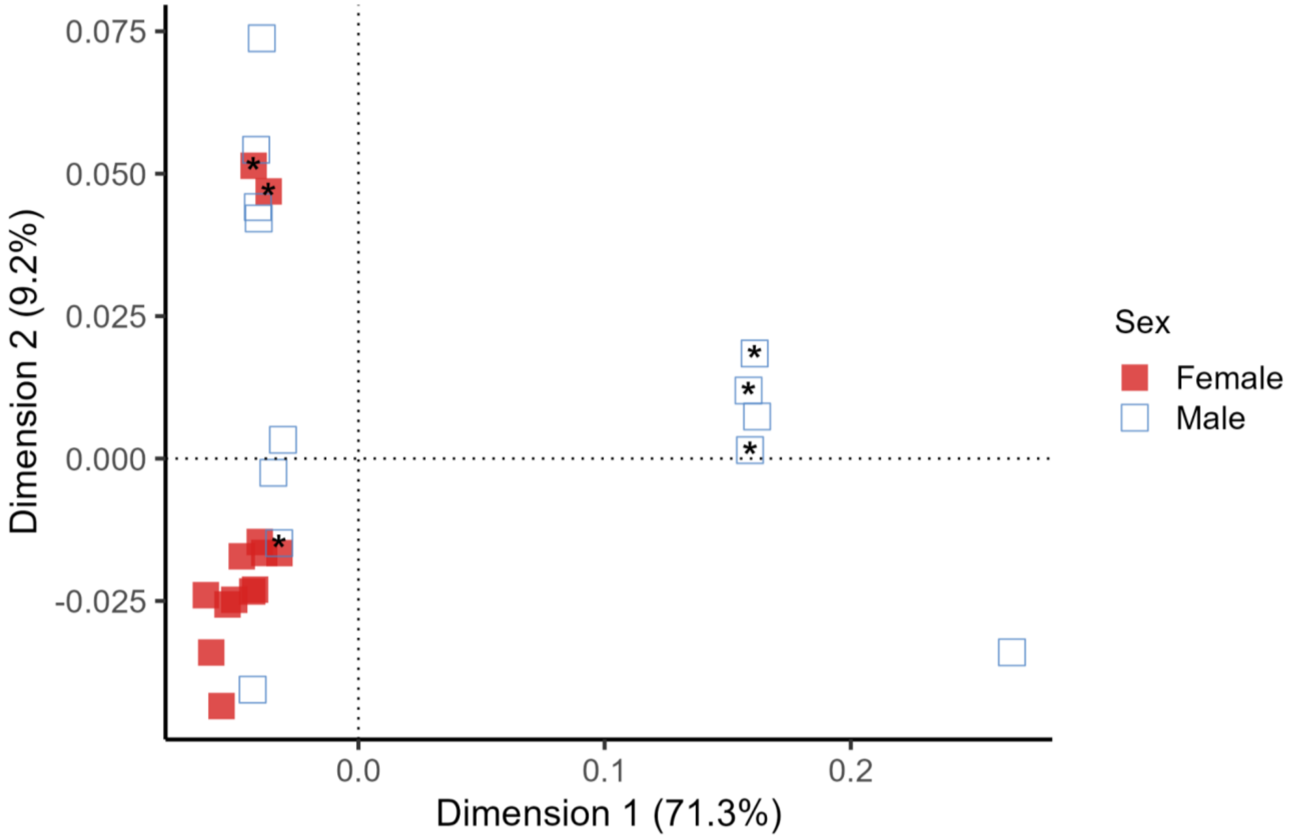

Figure S8. Clustering of 225 SNPs according to phenotypic sex within a sex-associated region (21.27 to 21.28 Mb) on chromosome 12 in *P. phoxinus.* Phenotypic sex was inferred by visual inspection of the gonads. The * symbol indicates samples from the Meuse population of *P. phoxinus.* In contrast to Figure S7 above, this region appears to be particularly restricted to the Meuse population.

Supplementary Figure S9: *P. phoxinus* Haplotype Phylogeny (Chromosome 3)

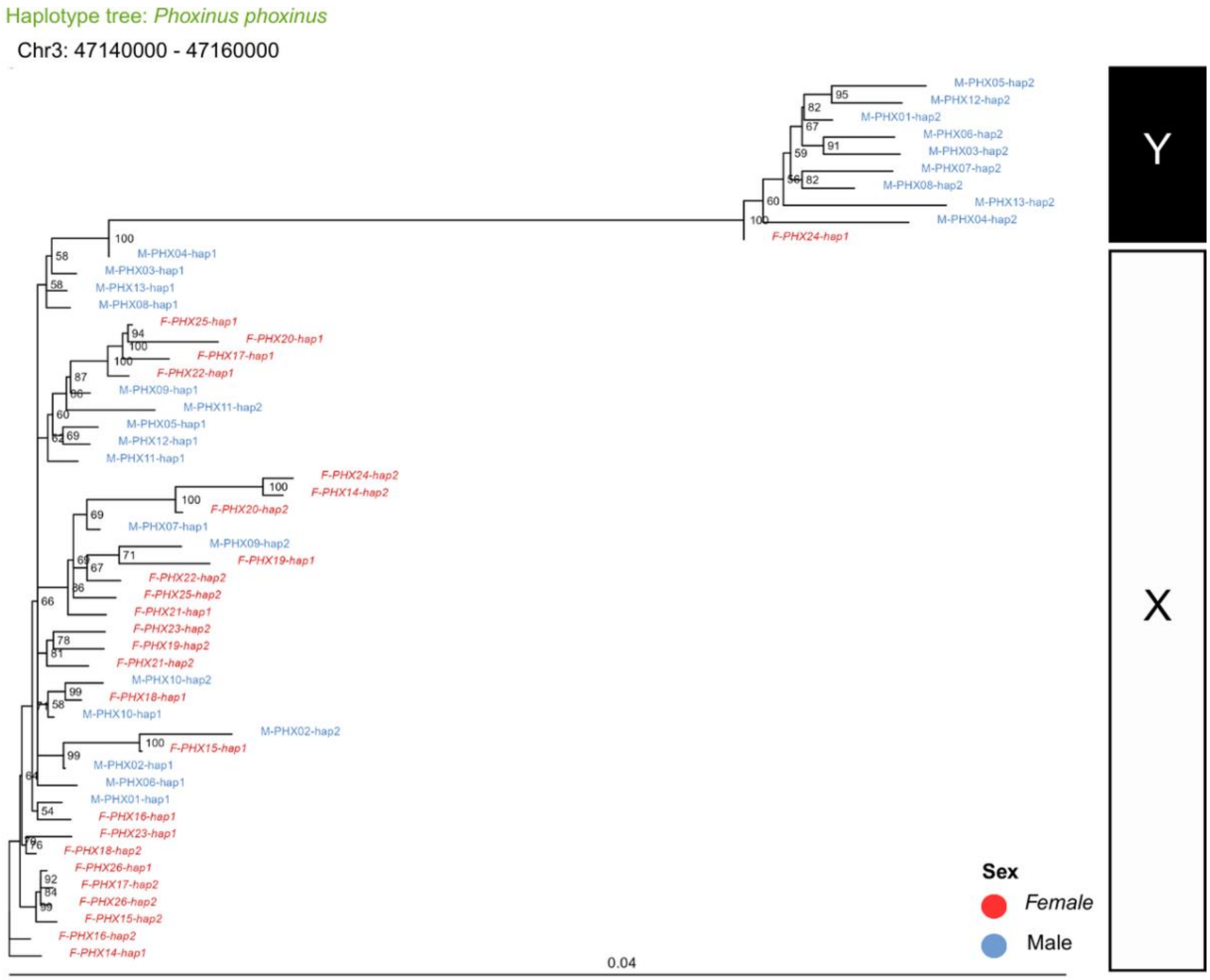

Figure S9. Haplotype phylogeny of the Sex-associated region (47.14–47.16 Mb) on chromosome 3 in *P. phoxinus*, based on consensus sequences from phased SNPs and reference positions, shows a clear difference between the Y haplotype in 9 of 13 males and the X haplotypes found in both males and females. The X haplotypes include single copies in males and double copies in females, supporting an XY sex-determination system. Y-linked sequences also appear to be strongly diverged from X-linked sequences.

Supplementary Figure S10: *P. phoxinus* Haplotype Phylogeny (Chromosome 12)

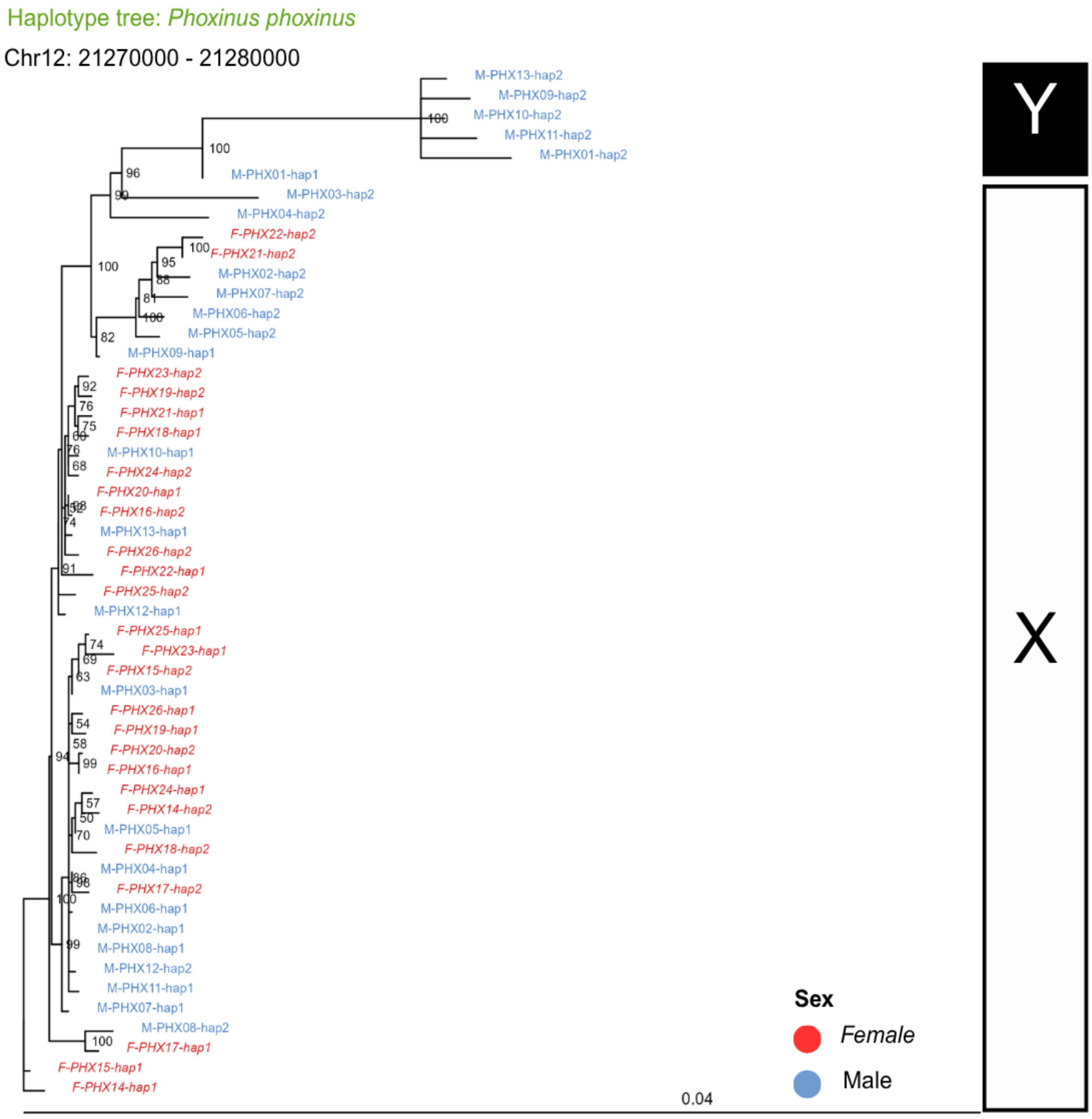

Figure S10. Haplotype phylogeny of the Sex-associated region (21.27–21.28 Mb) on chromosome 12 in *P. phoxinus*, based on consensus sequences, shows divergence between the Y haplotype in 5 of 13 males and the X haplotypes found in those males and all other individuals. The X haplotypes include single copies in males and double copies in females, thus supporting an XY sex-determination system. Y-linked sequences also appear to be strongly divergent from X-linked sequences; however, this divergence does not appear to be as pronounced as that observed on chromosome 3.

Supplementary Figure S11: *P. phoxinus* Linkage Block (Chromosome 3)

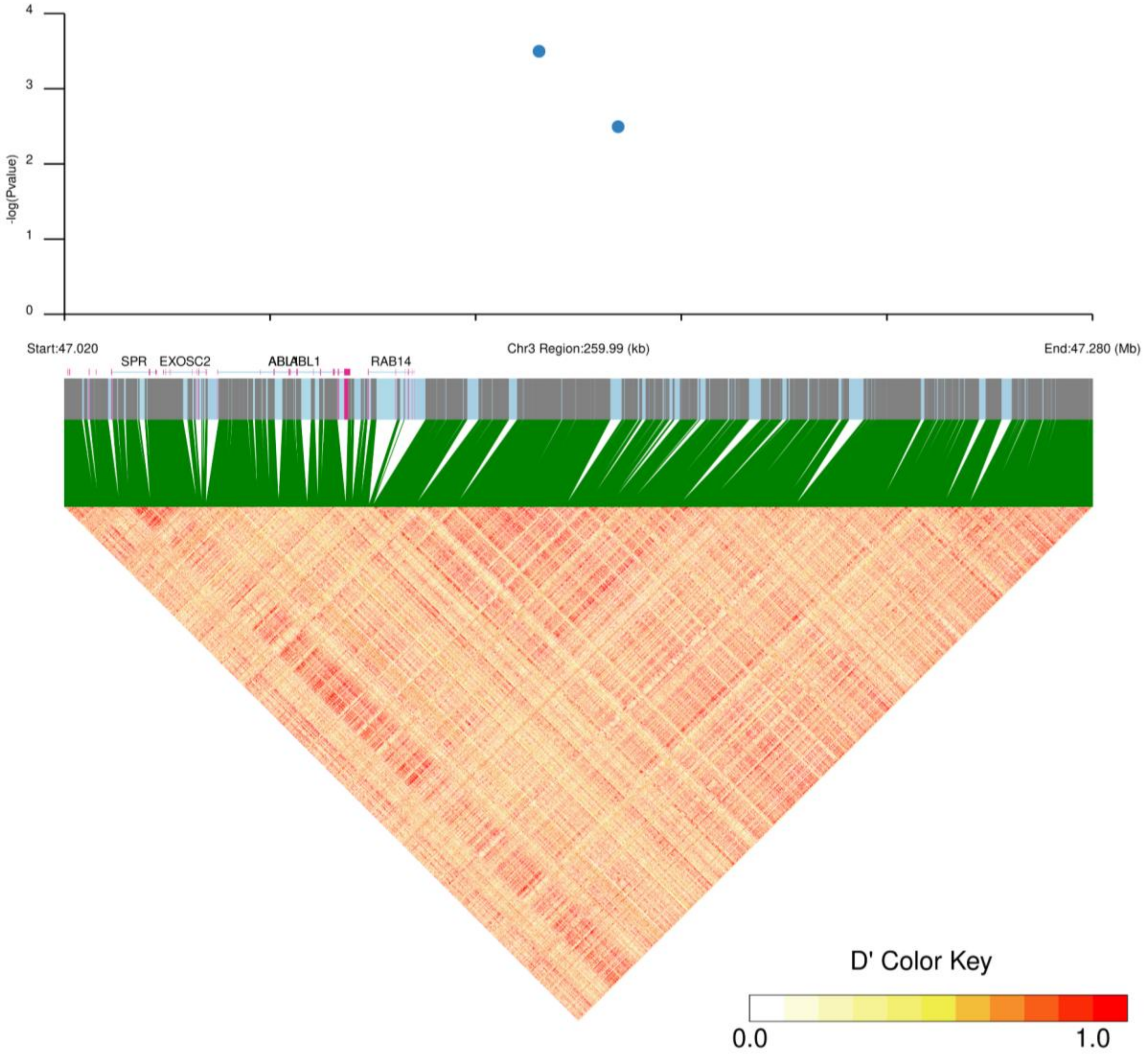

Figure S11. Linkage disequilibrium (LD) block around the sex-associated region (47.14–47.16 Mb) on chromosome 3 in *P. phoxinus*. Darker colours indicate stronger LD (measured by r^2 and normalised D′, where D′ = 1 denotes complete LD and D′ = 0 linkage equilibrium). Gene models and Sex-Associated Regions are shown above the LD plots, respectively.

Supplementary Figure S12: *P. phoxinus* Gene Ontology (Chromosome 3)

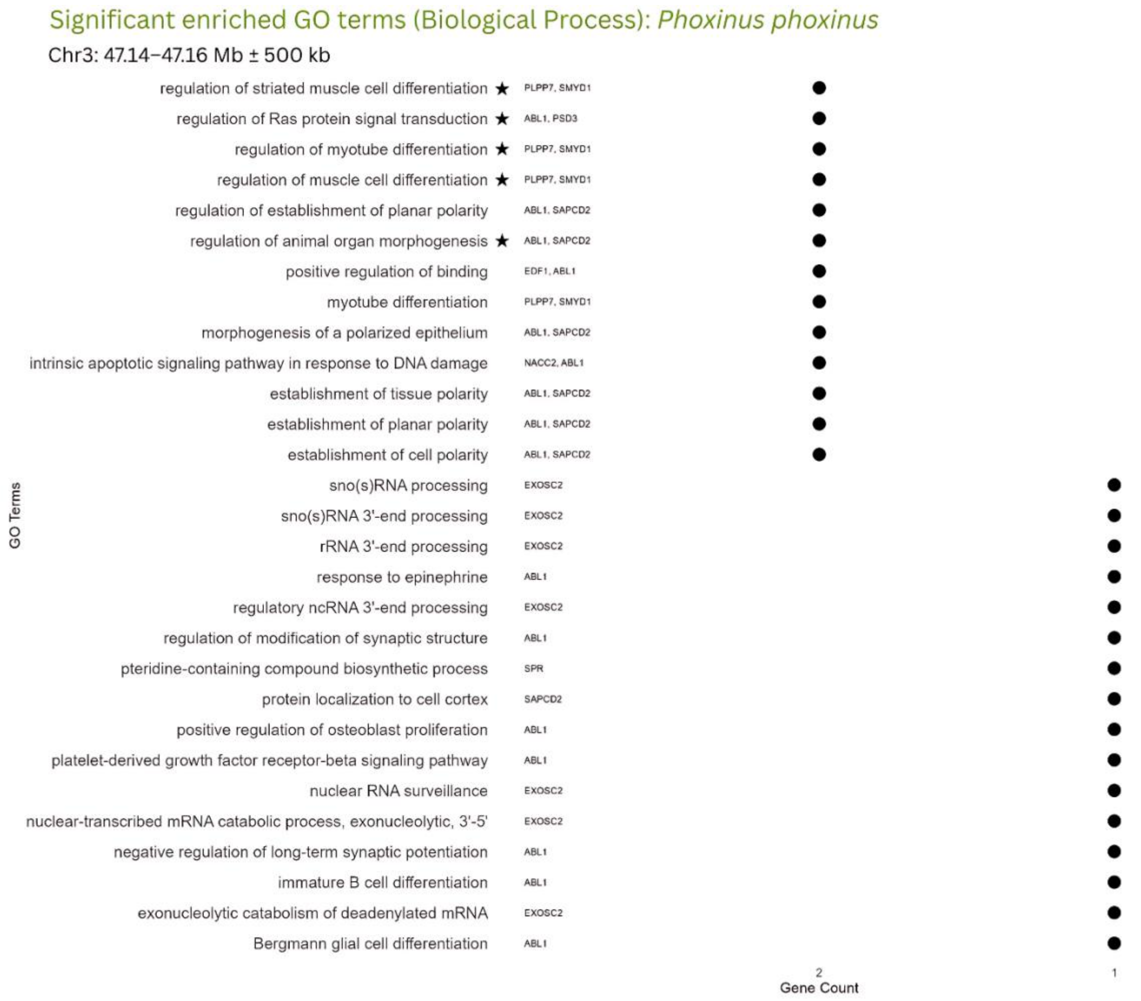

Figure S12. Gene Ontology (GO) analysis of biological processes associated with genes identified in the sex-associated region (47.14 to 47.16 Mb) on chromosome 3 in ***P. phoxinus***. Stars indicate terms directly linked to sex determination and differentiation or indirectly to gonadal development.

Supplementary Figure S13: P. phoxinus Linkage Block (Chromosome 12)

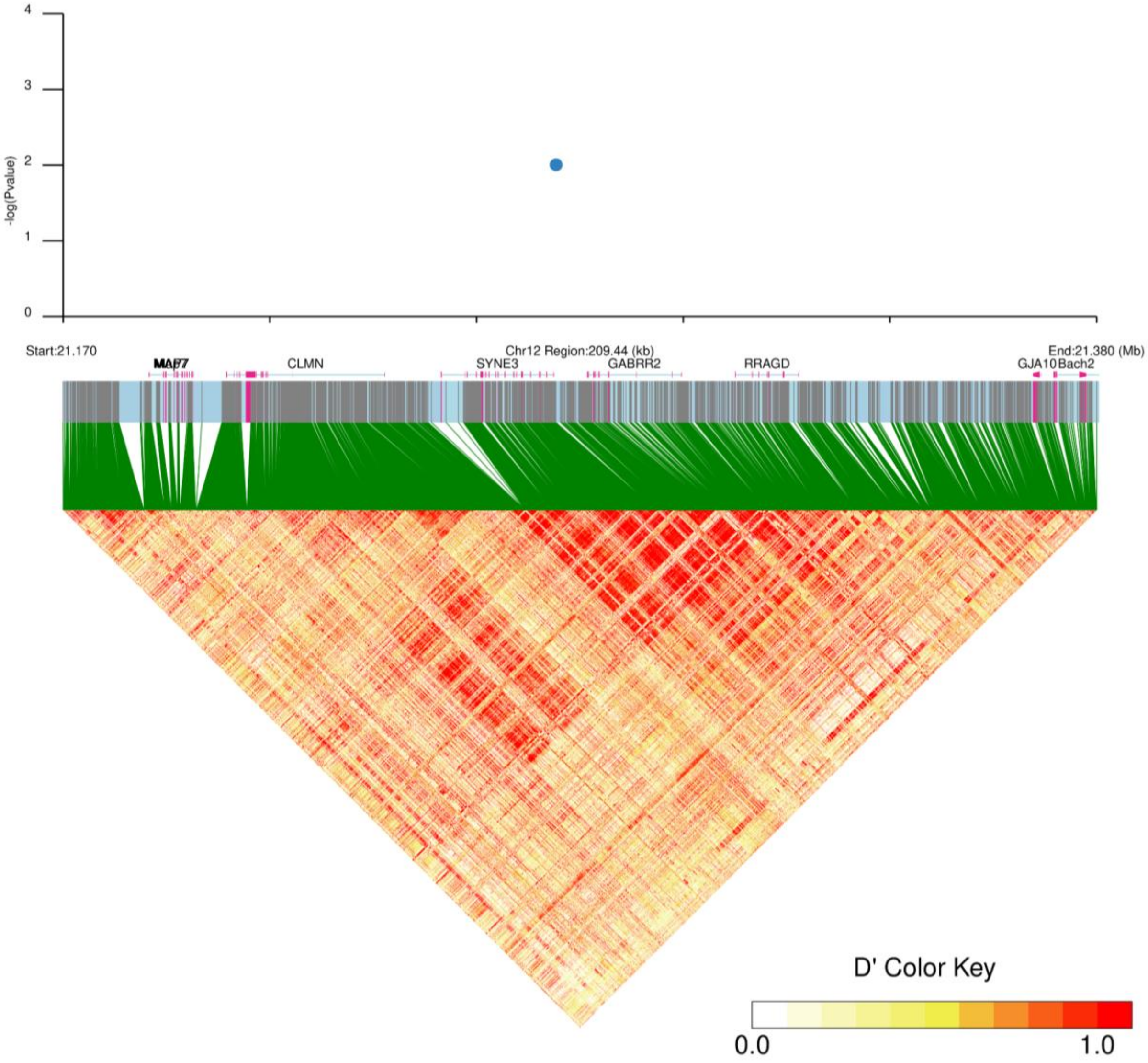

Figure S13. Linkage disequilibrium (LD) block around the sex-associated region (21.27–21.28 Mb) on chromosome 12 in *P. phoxinus*. Darker colours indicate stronger LD (measured by r^2 and normalised D′, where D′ = 1 denotes complete LD and D′ = 0 linkage equilibrium). Gene models and Sex-associated region are shown above the LD plots, respectively.

Supplementary Figure S14: *P. phoxinus* Gene Ontology (Chromosome 12)

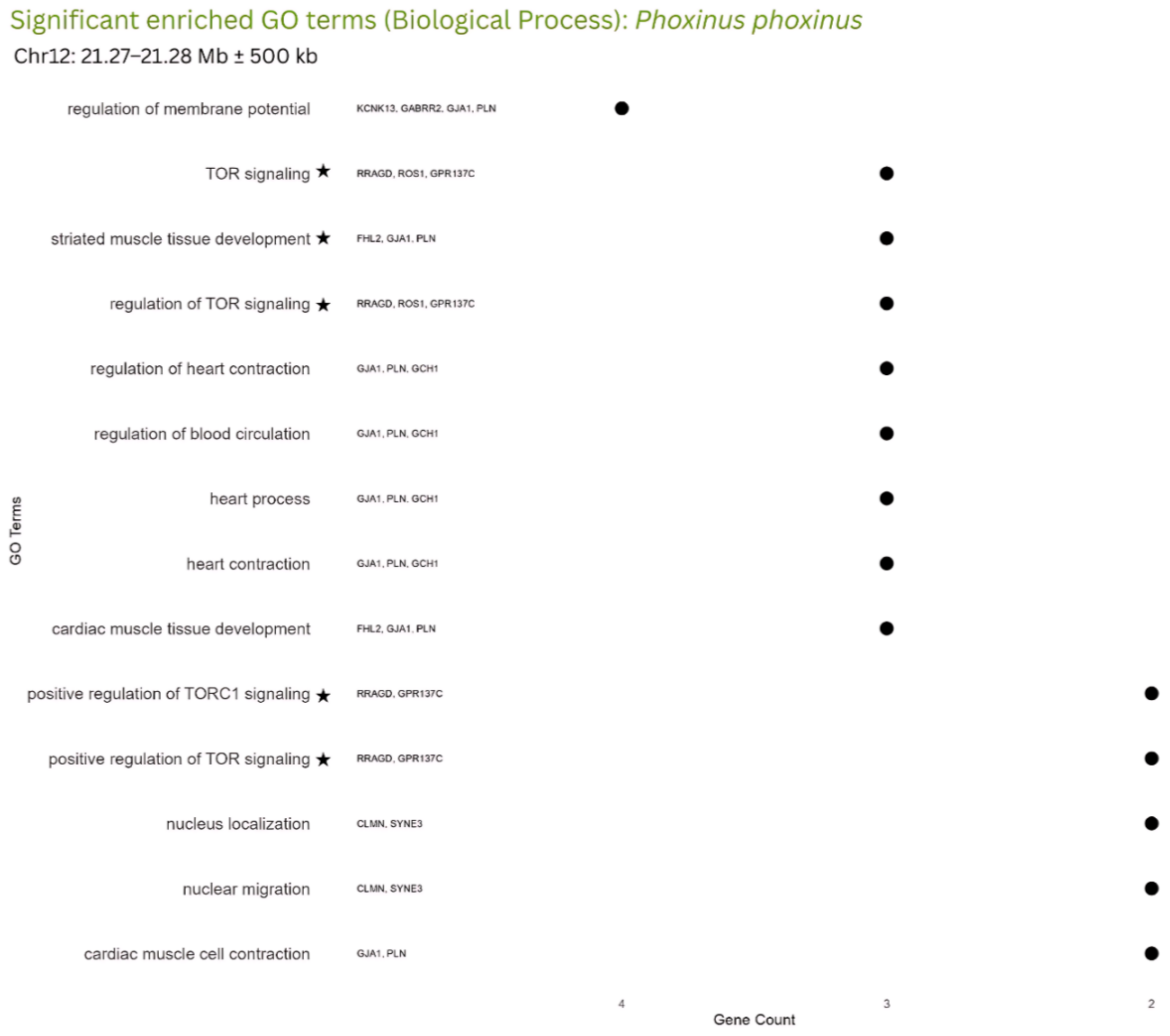

Figure S14. Gene Ontology (GO) analysis of biological processes associated with genes identified in the sex-associated region (21.27 to 21.28 Mb) on chromosome 12 in ***P. phoxinus***. Stars indicate GO terms directly linked to sex determination and differentiation or indirectly to gonadal development.

Supplementary Figure S15: ***P. csikii*** **Difcover Result.**

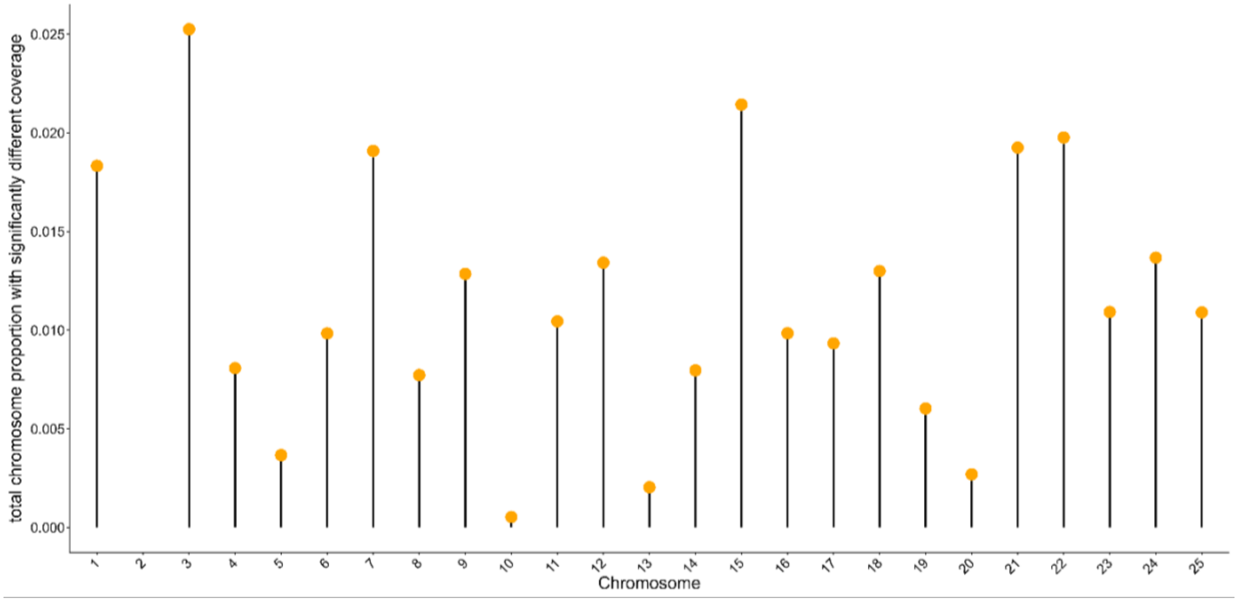

Figure S15. Coverage analysis reveals no highly differentiated sex chromomosomes between males and females in *P. csikii*. highlighting the proportion of local differences in coverage between male and female samples in *Phoxinus csikii*. Chromosomes 3 and 15 show the most pronounced differences in coverage, with a maximum of 2.5% of their chromosome length differing between the sexes. No region on chromosome 2 showed significant differences in coverage between males and females.

Supplementary Figure S16: *P. csikii* SNP-based Results

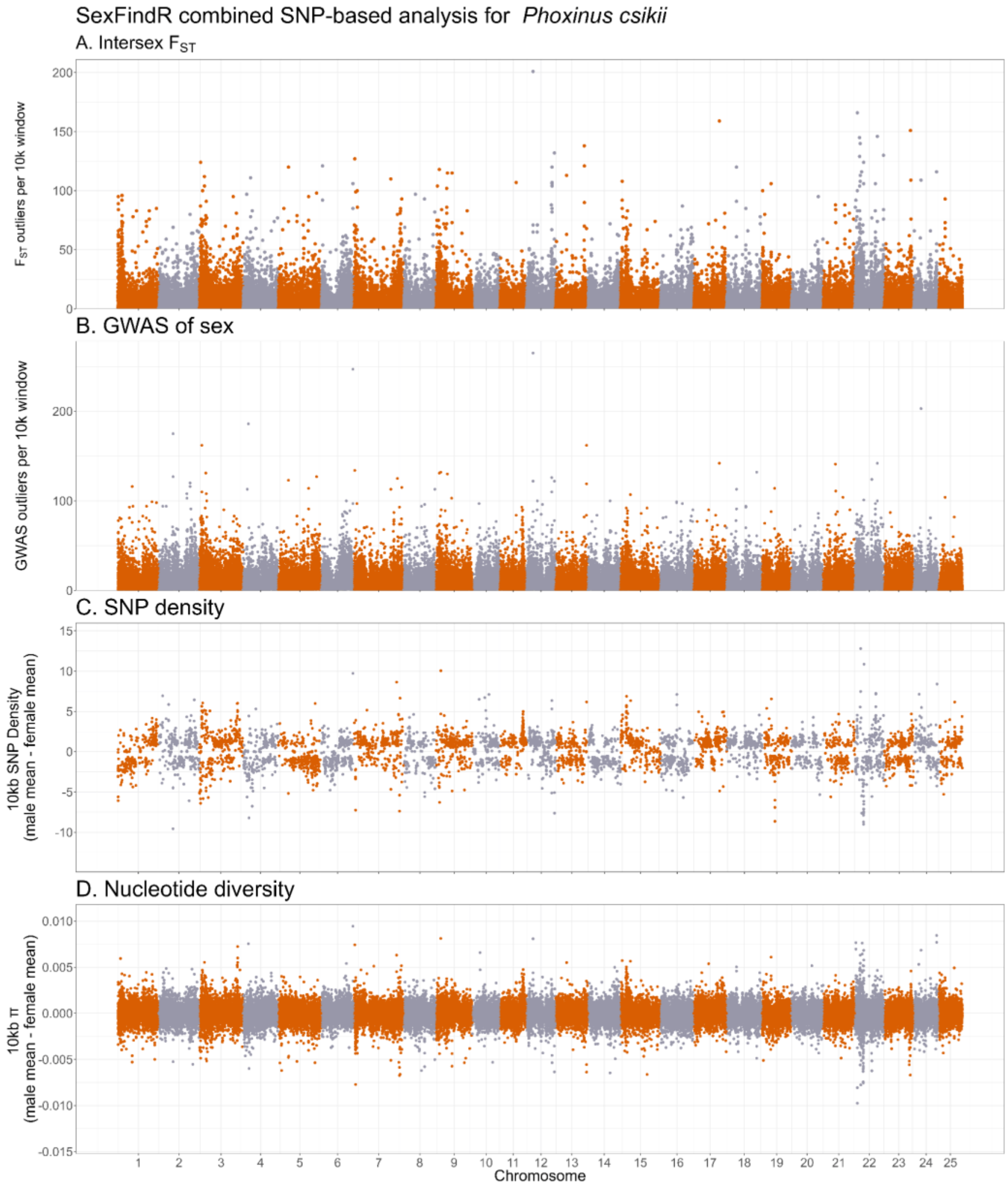

Figure S16. Results from four SNP-based analyses: F_{ST}, GWAS, SNP density, and nucleotide diversity—arranged sequentially from top to bottom. No section was associated with phenotypic sex. These analyses utilise approximately 18 million SNPs, evaluated in 10 kb windows for *P. csikii*. The absence of red lines indicates no overlapping signals across the analyses, identifying no potential sex-associated regions. However, interesting signals appear on Chromosome 22.

Supplementary Figure S17: *P. csikii* randomised-sex SNP-based Results

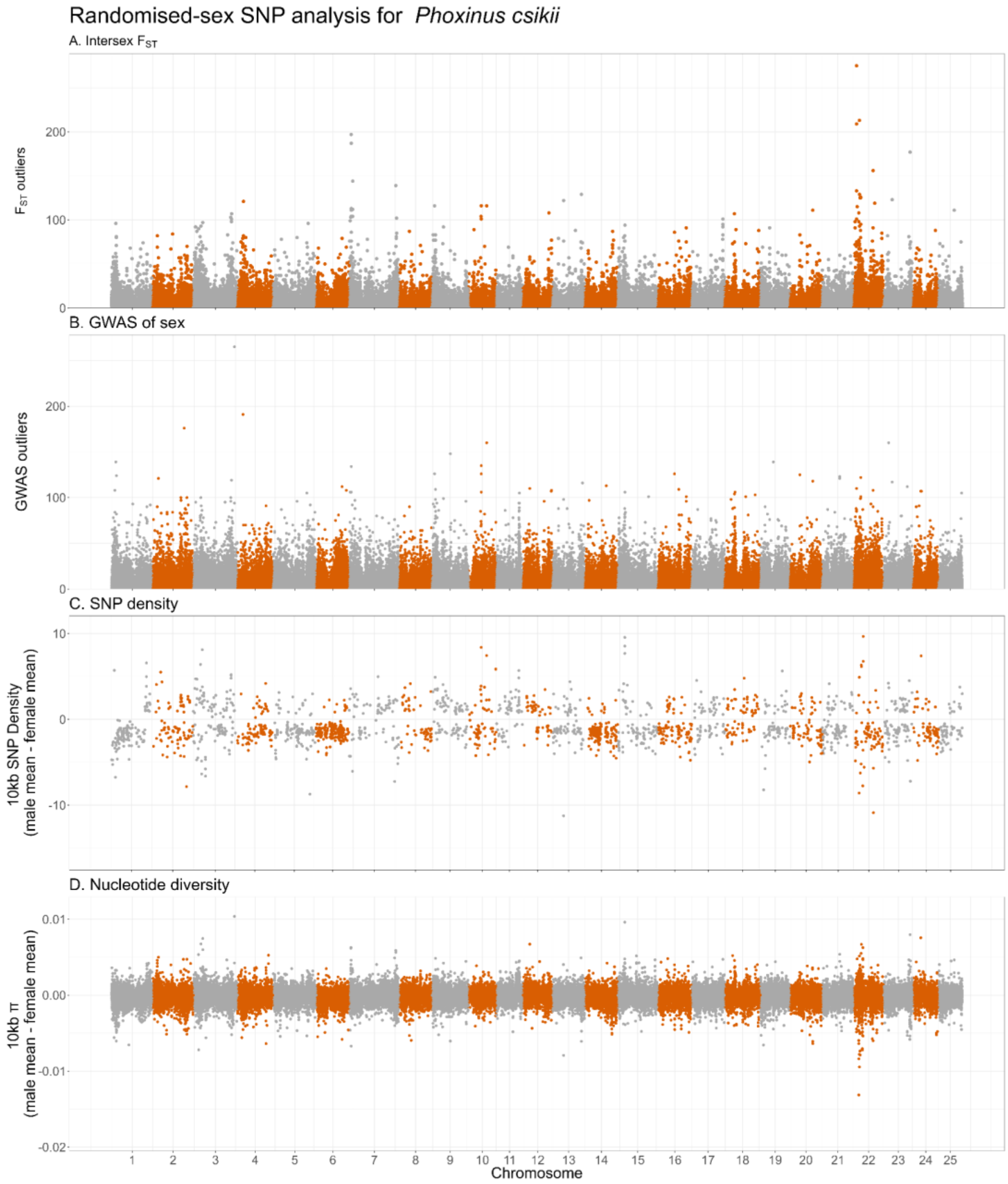

Figure S17. Results from four SNP-based analyses on randomised phenotypic sex labels — F_{ST}, GWAS, SNP density, and nucleotide diversity — are arranged from top to bottom. These analyses are based on approximately 18 million single-nucleotide polymorphisms (SNPs), evaluated in 10-kilobase windows, for P. csikii. It is evident that the signals on chromosome 22 have remained, thus indicating the presence of other non-sex-linked variations.

Supplementary Figure S18: *P. csikii* MDS (Full SNPs dataset)

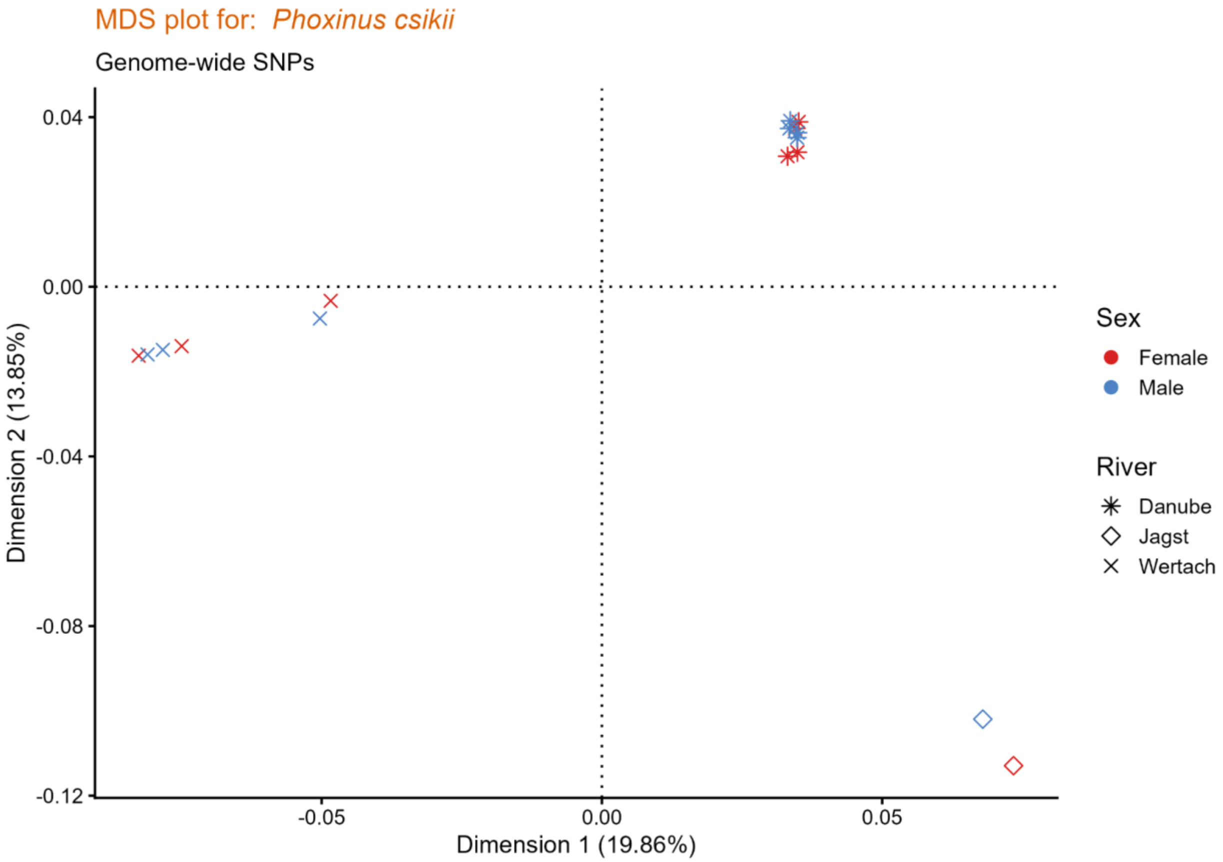

Figure S18. Multidimensional scaling (MDS) of ~18 million genome-wide SNPs in *P. phoxinus* from the Danube, Wertach, and Jagst drainages. The plot shows the first two MDS dimensions based on pairwise genetic distances. Diamond-shaped points represent samples from the Jagst, asterisk-shaped points represent Danube samples, and cross-shaped points represent samples from the Wertach. Individuals are observed to cluster according to the sampled drainage population rather than exhibiting any sex-linked patterns on a genome-wide scale.

Supplementary Figure S19: *P. csikii* MDS (Chromosome 3)

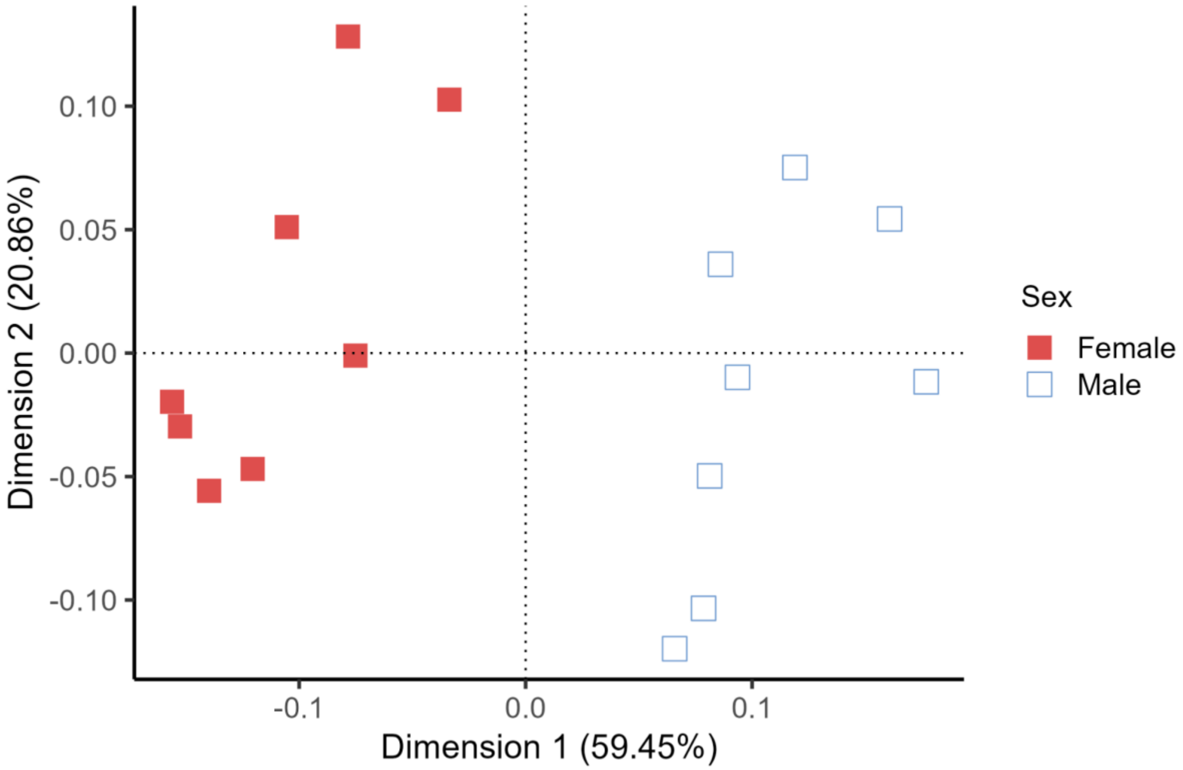

Figure S19. Multidimensional Scaling (MDS) Analysis of Sex-associated region in *P. csikii* chromosome 3 from 1,492,997 to 1,493,793 bases, comprising 76 SNPs, reveals clear differentiation between all male and female samples in this region, suggesting strong sex-association in this region.

Supplementary Figure S20: *P. csikii* Haplotype-phased Phylogeny (Chromosome 3)

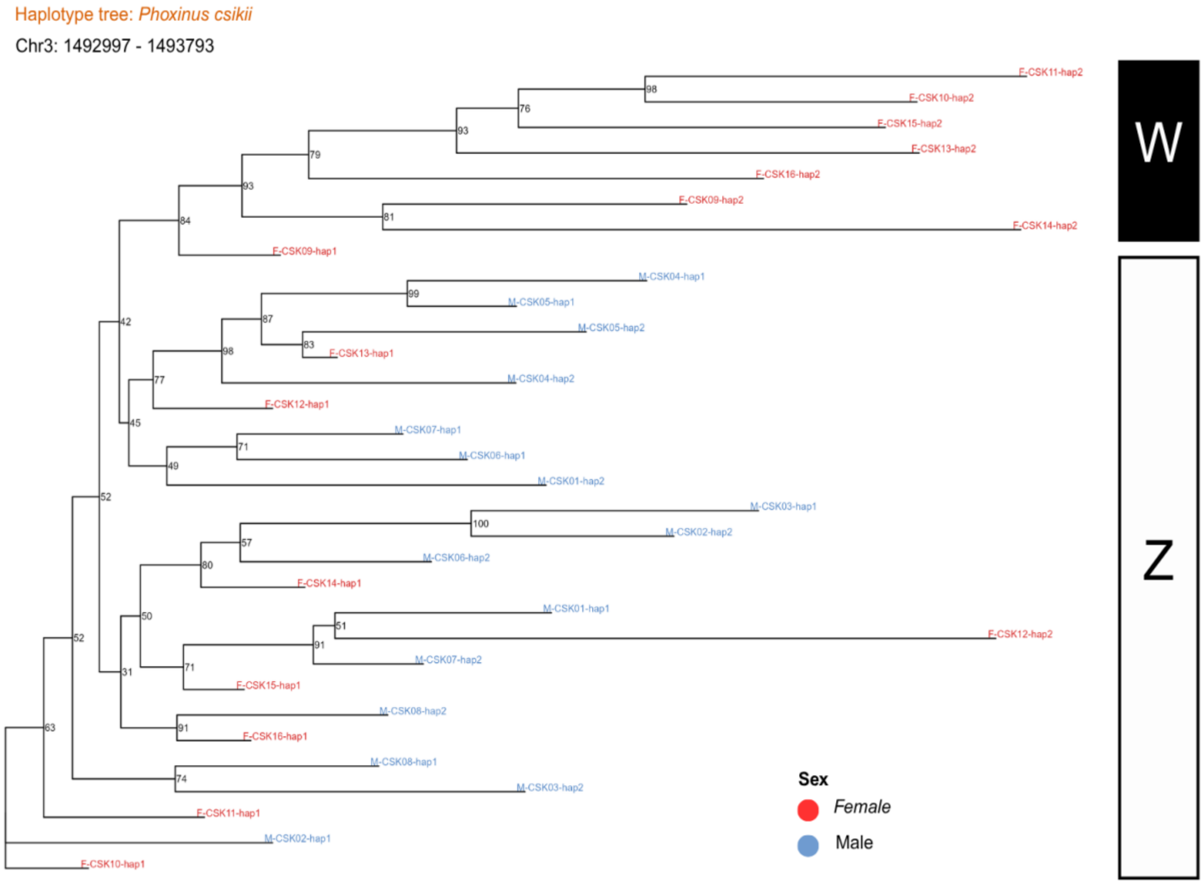

Figure S20. Haplotype phylogeny of the sex-associated region (1.49–1.49 Mb) on chromosome 3 in *P. csikii*, based on consensus sequences, shows a moderate divergence between the W haplotype in all 8 females and the Z haplotype. The Z haplotypes include single copies in females and double copies in males, supporting a ZW sex-determination system.

Supplementary Figure S21: *P. csikii* Linkage Block (Chr3)

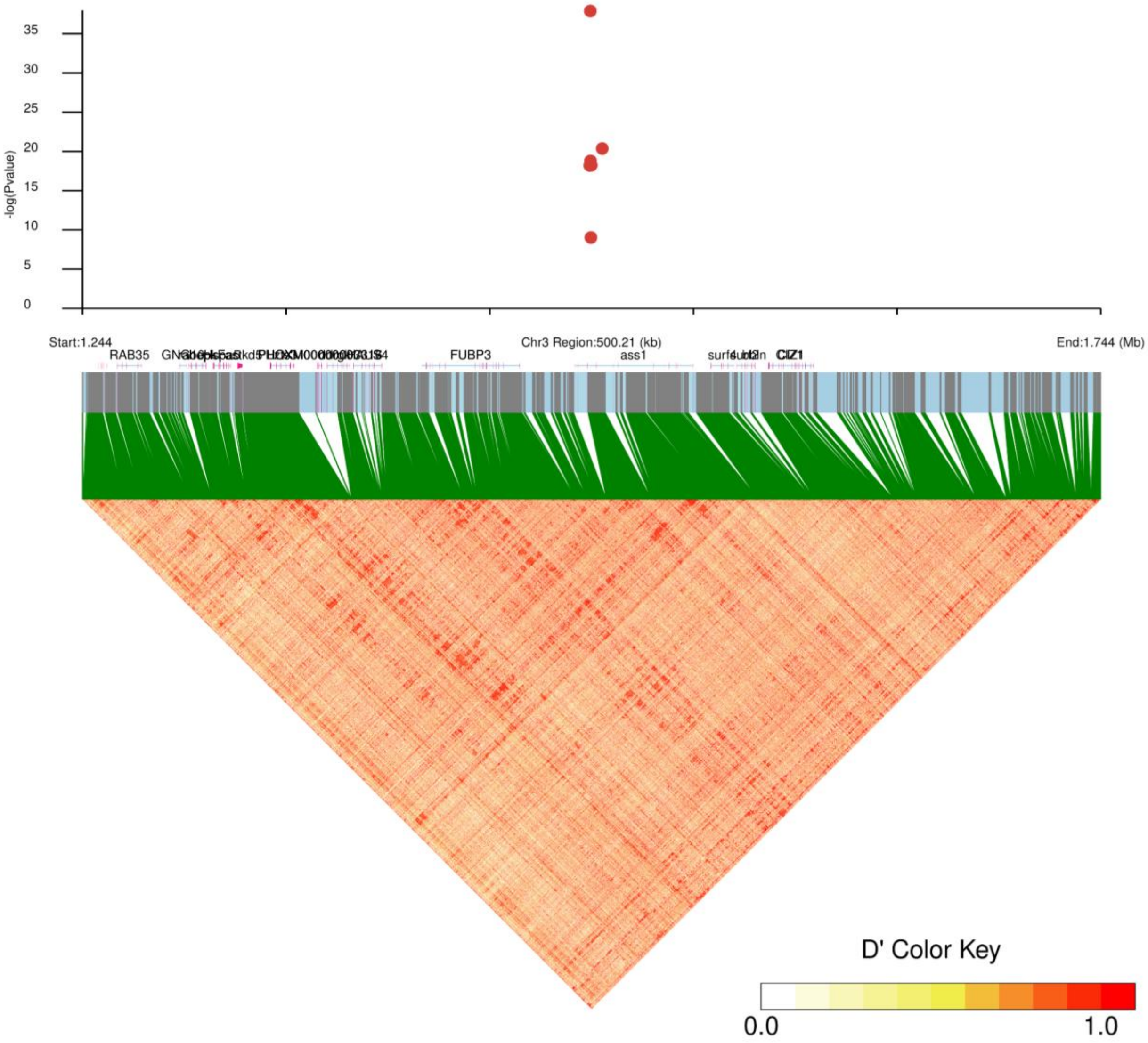

Figure S21. Linkage disequilibrium (LD) block around the sex-associated region (1.492–1.499 Mb) on chromosome 3 in *P. csikii*. Darker colors indicate stronger LD (measured by r^2 and normalised D′, where D′ = 1 denotes complete LD and D′ = 0 linkage equilibrium). Gene models and Sex-Associated Regions are shown above the LD plots, respectively.

Supplementary Figure S22: ***P. csikii*** **Gene Ontology (Chr3)**

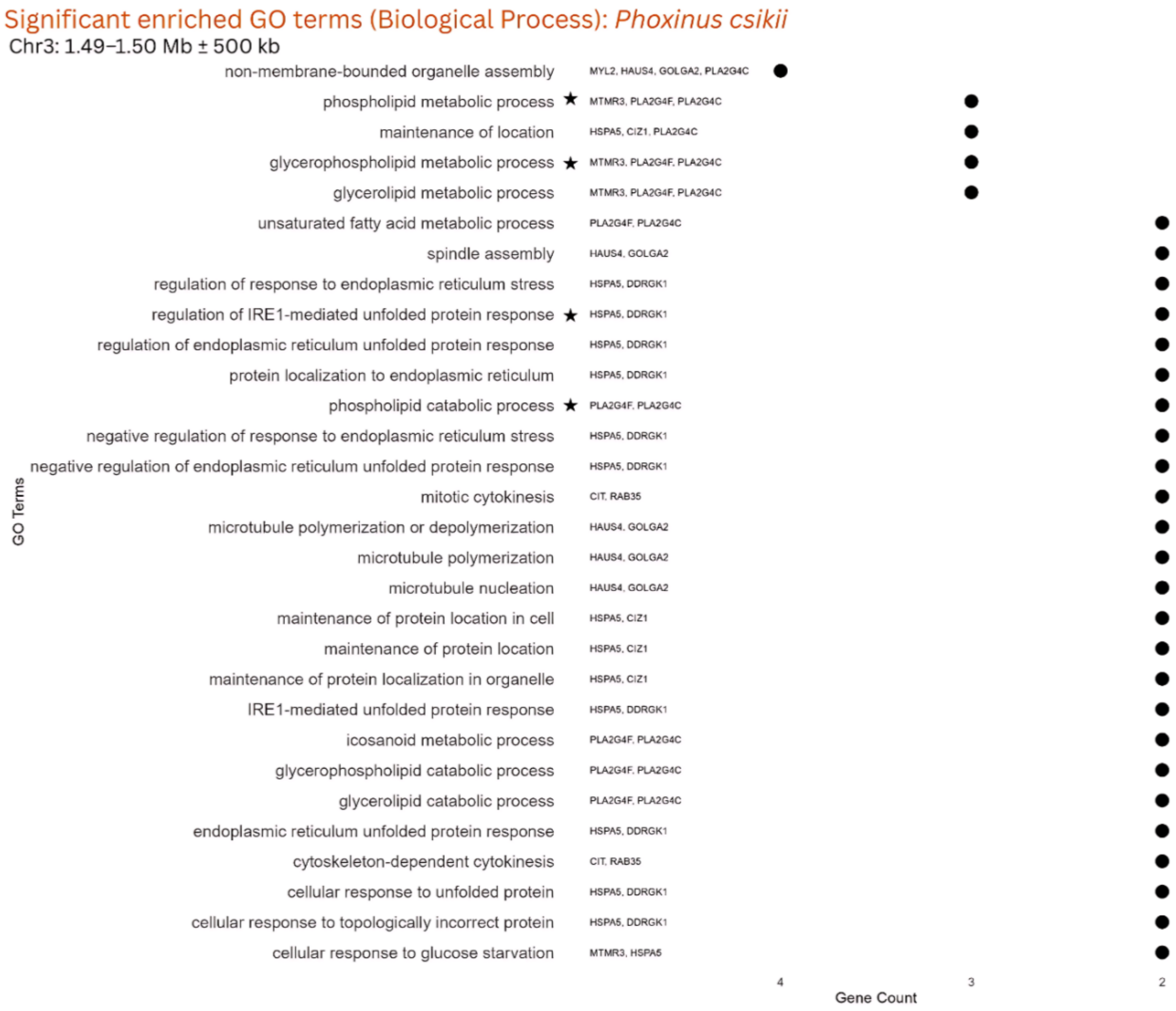

Figure S22. Gene Ontology (GO) analysis of biological processes associated with genes located near the candidate sex-associated region on Chr3: 1.49 Mb ±500 kb in *P. csikii*. Stars indicate GO terms directly linked to sex determination and differentiation or indirectly involved in gonadal development.

Supplementary Figure S23: *P. csikii* Gene Ontology (Chr 3)

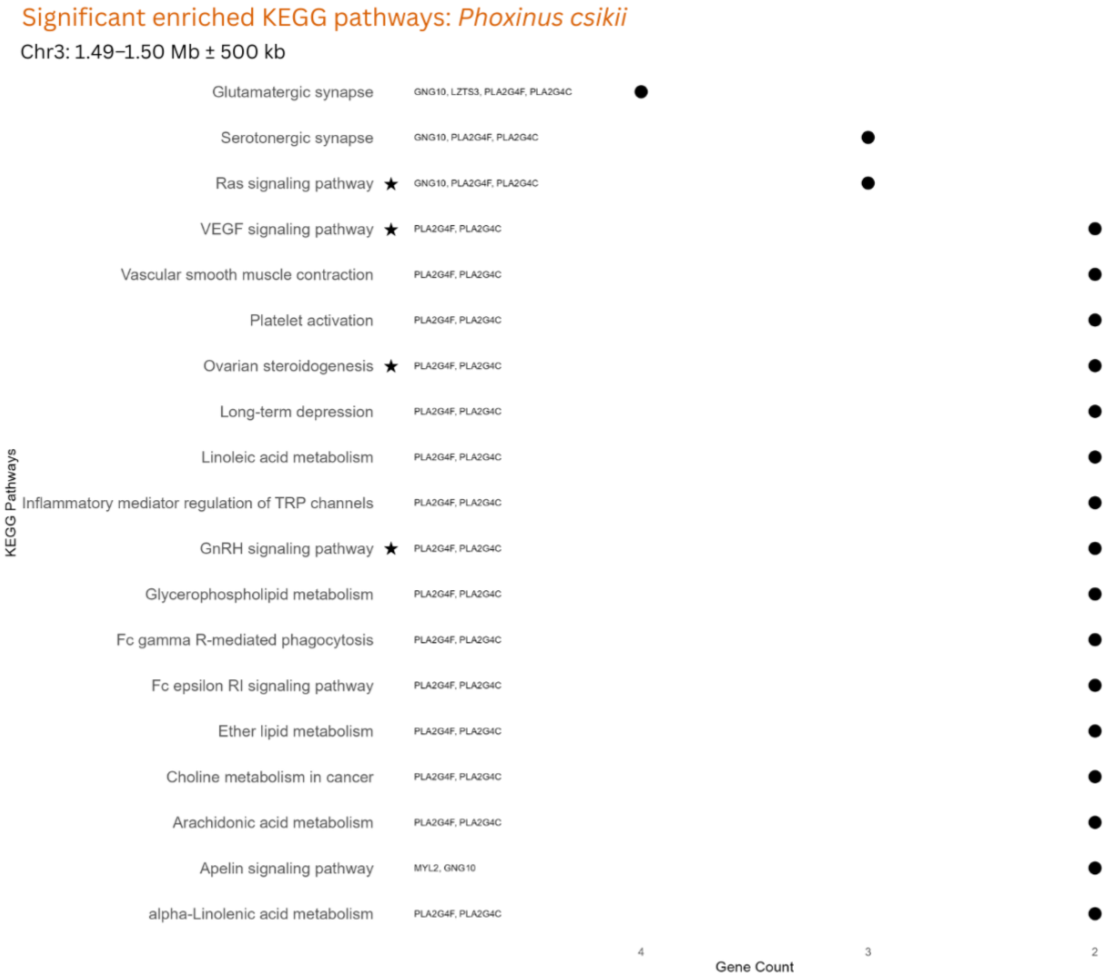

Figure S23. KEGG pathways associated with genes located near the candidate sex-associated region on Chr3: 1.49 Mb ±500 kb. Stars indicate KEGG pathways directly linked to sex determination and differentiation or indirectly involved in gonadal development.

Supplementary Table S2: Summary of Sex-associated Coverage by chromosome in *Phoxinus phoxinus*

Table S2: Summary of sex-associated coverage by chromosome in *P. phoxinus*. The table lists chromosome ID, the length of regions showing significant coverage differences between males and females (in base pairs), total chromosome length (in base pairs), and the percentage of the chromosome covered by these regions.

Chromosome	Total chromosome length (bp)	Sex-associated coverage (bp)	% sex-associated coverage
1	45,191,066	897,103	0.020
2	44,890,755	857,448	0.019
3	48,474,465	2,621,374	0.054
4	39,047,206	646,678	0.017
5	47,672,159	877,412	0.018
6	36,381,605	366,907	0.010
7	54,986,461	361,352	0.007
8	35,750,743	1,801,449	0.050
9	40,134,292	1,120,209	0.028
10	30,694,596	764,935	0.025
11	29,429,680	313,187	0.011
12	32,232,413	468,195	0.015
13	36,012,166	590,934	0.016
15	44,260,849	2,704,983	0.061
16	37,018,565	1,309,291	0.035
17	36,320,318	639,718	0.018
18	39,332,310	292,880	0.007
19	32,504,701	420,055	0.013
20	35,231,784	955,630	0.027
21	35,024,062	1,069,363	0.031
22	32,517,511	1,384,103	0.043
23	32,836,095	323,943	0.010
24	27,735,089	430,682	0.016
25	26,155,947	758,343	0.029

Supplementary Table S3: Genomic Regions Highlighted by SNP Density, F_{ST}, GWAS, and Nucleotide Diversity Rankings in *P. phoxinus*

Table S3: Candidate genomic windows identified by the combined ranking of four sex-associated metrics in *P. phoxinus*. Windows were ranked based on SNP density differences between males and females, intersex F_{ST} outlier counts (F_{ST} rank), sex-associated GWAS outlier counts (GWAS rank), and nucleotide diversity differences (π rank). The table lists chromosome number, genomic start and end position, Chromosome length, and ranks for each metric. Candidate windows represent the top 100-ranked windows for each metric, highlighting genomic regions potentially involved in sex determination or sex-specific differentiation.

Chromosome	Start	End	Length	SNP density rank	F_{ST} rank	GWAS rank	π rank
3	47140000	47150000	48474465	5	10	7	35
3	47150000	47160000	48474465	12	21	27	64
12	21270000	21280000	32232413	6	65	59	22

Supplementary Table S4: Genes Located 500 kb Upstream and Downstream of the *P. phoxinus* Candidate Sex-associated region on Chromosome 3

Table S4. List of candidate genes located within 500 kb upstream and downstream of the *P. phoxinus* candidate sex-associated region on chromosome 3 (Chr3: 47,14 Mb ± 500 kb).

No.	Position	Gene Symbol	Gene Name
1	Chr3:46565966-46566020	*GPSM1*	G-Protein Signalling Modulator 1
2	Chr3:46682317-46689499	*TMEM250*	Transmembrane Protein 250
3	Chr3:46729028-46738343	*NACC2*	Nucleus Accumbens Associated 2
4	Chr3:46749493-46822143	*NUP214*	Nucleoporin 214
5	Chr3:46833931-46844995	*FAM78A*	Family with Sequence Similarity 78 Member A
6	Chr3:46857406-46859517	*PLPP7*	Phospholipid Phosphatase 7
7	Chr3:46876501-46918606	*PRRC2B*	Proline-Rich Coiled-Coil 2B
8	Chr3:46922970-46932250	*EDF1*	Endothelial Differentiation-Related Factor 1
9	Chr3:46937434-46953332	*DQX1*	Deaq-Box RNA-Dependent ATPase 1
10	Chr3:47009149-47011224	*FABP1A*	Fatty Acid Binding Protein 1
11	Chr3:47011701-47028090	*SMYD1*	SET and MYND Domain Containing 1
12	Chr3:47031780-47043341	*SPR*	Sepiapterin Reductase
13	Chr3:47044931-47056042	*EXOSC2*	Exosome Component 2
14	Chr3:47058678-47092278	*ABL1*	Non-Receptor Tyrosine Kinase
15	Chr3:47096714-47108511	*RAB14*	Ras-Related Protein Rab-14
16	Chr3:47529702-47558114	*PSD3*	Pleckstrin And Sec7 Domain Containing 3
17	Chr3:47589496-47625142	*SAPCD2*	Suppressor APC Domain Containing 2
18	Chr3:47653472-47654396	*P2RY3*	Purinergic Receptor P2Y3

Supplementary Table S5: Enriched Gene Ontology (GO) Terms Among Genes Adjacent to the *P. phoxinus* Sex-associated region on chromosome 3

Table S5. Significantly enriched GO terms among genes located near the candidate sex-associated region on chromosome 3 in *P. phoxinus* (Chr3: 47.14–47.16 Mb ±500 kb). Shown are GO terms with nominal $p < 0.01$, the number of genes overlapping each term, and the associated genes.

GO Term ID	Description	Gene Count	Gene Symbol	p-value
GO:0010830	Regulation of myotube differentiation	2	*PLPP7, SMYD1*	0.00047
GO:0090175	Regulation of the establishment of planar polarity	2	*ABL1, SAPCD2*	0.00059
GO:0001736	Establishment of planar polarity	2	*ABL1, SAPCD2*	0.00103
GO:0007164	Establishment of tissue polarity	2	*ABL1, SAPCD2*	0.00103
GO:0001738	Morphogenesis of a polarised epithelium	2	*ABL1, SAPCD2*	0.00177
GO:0051153	Regulation of striated muscle cell differentiation	2	*PLPP7, SMYD1*	0.00197
GO:0008630	Apoptotic signalling in response to DNA damage	2	*NACC2, ABL1*	0.00217
GO:2000027	Regulation of animal organ morphogenesis	2	*ABL1, SAPCD2*	0.00329
GO:0051099	Positive regulation of binding	2	*EDF1, ABL1*	0.00334
GO:0014902	Myotube differentiation	2	*PLPP7, SMYD1*	0.00350
GO:0030010	Establishment of cell polarity	2	*ABL1, SAPCD2*	0.00499
GO:0051147	Regulation of muscle cell differentiation	2	*PLPP7, SMYD1*	0.00537
GO:0046578	Regulation of Ras protein signal transduction	2	*ABL1, PSD3*	0.00680
GO:0031125	rRNA 3'-end processing	1	*EXOSC2*	0.00687
GO:0060020	Bergmann glial cell differentiation	1	*ABL1*	0.00755
GO:0072697	Protein localisation to cell cortex	1	*SAPCD2*	0.00755
GO:0002327	Immature B cell differentiation	1	*ABL1*	0.00824
GO:0033690	Positive regulation of osteoblast proliferation	1	*ABL1*	0.00892
GO:0042559	Pteridine biosynthetic process	1	*SPR*	0.00892
GO:1900272	Negative regulation of synaptic potentiation	1	*ABL1*	0.00892

GO:1905244	Regulation of synaptic structure modification	1	*ABL1*	0.00892
GO:0035791	PDGF receptor-beta signalling pathway	1	*ABL1*	0.00961
GO:0071871	Response to epinephrine	1	*ABL1*	0.00961

Supplementary Table S6: Genes Located 500 kb Upstream and Downstream of the *P. phoxinus* Candidate Sex-associated region on Chromosome 12

Table S6: List of candidate genes located within 500 kb upstream and downstream of the *P. phoxinus* candidate sex-associated region on chromosome 12 (Chr12: 21.27 Mb ± 500 kb).

No.	Position	Gene Symbol	Gene Name
1	Chr12:20762790-20782177	*STON2*	Syntaxin-2
2	Chr12:20809493-20868948	*FOXN3*	Forkhead box N3
3	Chr12:20938578-21003883	*EFCAB11*	EFCAB domain-containing protein 11
4	Chr12:21005183-21036158	*TDP1*	Tyrosyl-DNA phosphodiesterase 1
5	Chr12:21040487-21043246	*KCNK13*	Potassium channel subfamily K member 13
6	Chr12:21051856-21057602	*HMBOX1*	Homeobox protein 1
7	Chr12:21073639-21115334	*KIF13B*	Kinesin family member 13B
8	Chr12:21156034-21161790	*PNOC*	Pro-neuropeptide Y C-terminal peptide
9	Chr12:21187454-21196653	*MAP7*	Microtubule-associated protein 7
10	Chr12:21203138-21235329	*CLMN*	Climanin protein
11	Chr12:21246635-21269593	*SYNE3*	Nesprin-3
12	Chr12:21276293-21295453	*GABRR2*	Gamma-aminobutyric acid receptor subunit R2
13	Chr12:21306248-21319221	*RRAGD*	Ras-related GTP-binding protein D
14	Chr12:21366609-21368376	*GJA10*	Gap junction protein alpha-10
15	Chr12:21370773-21380032	*BACH2*	BTB and CNC homology 2
16	Chr12:21394307-21397036	*MANEA*	Manea protein
17	Chr12:21411920-21413000	*FUT9*	Fucosyltransferase 9
18	Chr12:21426168-21429079	*FHLl2*	Four and a half LIM domains 2
19	Chr12:21430432-21437246	*TGFBRAP1*	TGF-beta receptor-associated protein 1
20	Chr12:21461256-21465054	*COL10A1*	Collagen alpha-1(X) chain
21	Chr12:21467705-21469234	*MARCKS*	Myristoylated alanine-rich C kinase substrate
22	Chr12:21475830-21485266	*FYNA*	Fyn-associated protein A
23	Chr12:21539400-21540258	*FABP7*	Fatty acid-binding protein 7
24	Chr12:21546607-21547672	*GJA1*	Gap junction protein alpha-1
25	Chr12:21549389-21557247	*SYTL2*	Synaptotagmin-like protein 2
26	Chr12:21559886-21622304	*FAM184A*	Family with sequence similarity 184 member A
27	Chr12:21625681-21625840	*PLN*	Phospholamban
28	Chr12:21640010-21656505	*ROS1*	Proto-oncogene tyrosine-protein kinase ROS

29	Chr12:21657504-21666449	*STYX-A*	Styx protein A
30	Chr12:21667967-21671130	*GNPNAT1*	Glucosamine-6-phosphate N-acetyltransferase 1
31	Chr12:21679295-21680441	*SOCS4*	Suppressor of cytokine signalling 4
32	Chr12:21682950-21700104	*WDHD1*	WD repeat-containing protein 1
33	Chr12:21701106-21711575	*GCH1*	GTP cyclohydrolase 1
34	Chr12:21718182-21756894	*SAMD4A*	SAM domain-containing protein 4A
35	Chr12:21760549-21764120	*ERO1A*	Endoplasmic reticulum oxidoreductase 1 alpha
36	Chr12:21769800-21793271	*GPR137C*	G-protein coupled receptor 137C

Supplementary Table S7: Enriched Gene Ontology (GO) Terms Among Genes Adjacent to the *P. phoxinus* Sex-associated region on chromosome 12

Table S7. Significantly enriched GO terms among genes located near the candidate sex-associated region on chromosome 12 in *P. phoxinus* (Chr12: 21.27 Mb ± 500 kb). Shown are GO terms with nominal $p < 0.01$, the number of genes overlapping each term, and the associated genes.

GO Term ID	Description	Gene Count	Gene Symbols	p-value
GO:0042391	Regulation of membrane potential	4	*KCNK13, GABRR2, GJA1, PLN*	0.006254573
GO:0032006	Regulation of TOR signalling	3	*RRAGD, ROS1, GPR137C*	0.001946809
GO:0031929	TOR signaling	3	*RRAGD, ROS1, GPR137C*	0.002796608
GO:0008016	Regulation of heart contraction	3	*GJA1, PLN, GCH1*	0.005174011
GO:0060047	Heart contraction	3	*GJA1, PLN, GCH1*	0.007942982
GO:0048738	Cardiac muscle tissue development	3	*FHL2, GJA1, PLN*	0.008305297
GO:0003015	Heart process	3	*GJA1, PLN, GCH1*	0.009059041
GO:1903522	Regulation of blood circulation	3	*GJA1, PLN, GCH1*	0.009351742
GO:0014706	Striated muscle tissue development	3	*FHL2, GJA1, PLN*	0.009953711
GO:0007097	Nuclear migration	2	*CLMN, SYNE3*	0.000815698
GO:0051647	Nucleus localization	2	*CLMN, SYNE3*	0.001423529
GO:1904263	Positive regulation of TORC1 signalling	2	*RRAGD, GPR137C*	0.003503947
GO:0032008	Positive regulation of TOR signalling	2	*RRAGD, GPR137C*	0.006089124
GO:0086003	cardiac muscle cell contraction	2	*GJA1, PLN*	0.007342922

Supplementary Table S8: Summary of Sex-associated Coverage by chromosome in *Phoxinus csikii*

Table S8. Summary of sex-associated coverage by chromosome in *P. csikii*. The table lists chromosome ID, the length of regions showing significant differential coverage between males and females (in base pairs), total chromosome length (in base pairs), and the ratio of chromosome length covered by these regions.

Chromosome	Total chromosome	Sex-associated	% sex-associated
1	45.191.066	828.305	0.018
3	48.474.465	1.224.073	0.025
4	39.047.206	315.452	0.008
5	47.672.159	174.814	0.003
6	36.381.605	357.926	0.010
7	54.986.461	1.049.564	0.019
8	35.750.743	276.095	0.008
9	40.134.292	515.735	0.013
10	30.694.596	16.265	0.001
11	29.429.680	307.491	0.010
12	32.232.413	432.584	0.013
13	36.012.166	73.453	0.002
14	35.931.519	286.150	0.008
15	44.260.849	948.676	0.021
16	37.018.565	364.374	0.010
17	36.320.318	338.966	0.009
18	39.332.310	511.215	0.013
19	32.504.701	196.037	0.006
20	35.231.784	94.897	0.003
21	35.024.062	674.348	0.019
22	32.517.511	642.721	0.020
23	32.836.095	358.630	0.011
24	27.735.089	379.250	0.014
25	26.155.947	285.152	0.011

Supplementary Table S9: Genes Located 500 kb Upstream and Downstream of the *P. csikii* Candidate Sex-associated region on Chromosome 3

Table S9: List of candidate genes located within 500 kb upstream and downstream of the *P. csikii* candidate sex-associated region on chromosome 3 (Chr3: 1.49 Mb ± 500 kb).

No.	Position	Gene Symbol	Gene Name
1	Chr3:1030433-1051035	*PPP1CC-A*	Protein phosphatase 1 catalytic subunit alpha
2	Chr3:1053807-1061106	*MYL2*	Myosin light chain 2
3	Chr3:1070611-1082679	*CUX1*	Cut-like homeobox 1
4	Chr3:1098404-1130251	*MTMR3*	Myotubularin-related protein 3
5	Chr3:1137125-1255845	*CIT*	Citron Rho-interacting serine/threonine kinase
6	Chr3:1260818-1272964	*RAB35*	Ras-related protein Rab-35
7	Chr3:1291422-1294915	*GNG10*	G-protein subunit gamma-10
8	Chr3:1295847-1304596	*RABEPK*	Rab GTPase-binding effector protein kinase
9	Chr3:1307988-1316605	*HSPA5*	Heat shock protein family A member 5
10	Chr3:1319930-1322303	*FASTKD5*	FAST kinase domain-containing protein 5
11	Chr3:1335923-1347708	*LZTS3*	Leucine zipper tumour suppressor 3
12	Chr3:1363770-1375074	*DDRGK1*	DDRGK domain containing 1
14	Chr3:1376739-1390922	*HAUS4*	HAUS augmin-like complex subunit 4
15	Chr3:1410330-1458664	*FUBP3*	Far upstream element-binding protein 3
16	Chr3:1485507-1543791	*ASS1*	Asparagine synthetase 1
17	Chr3:1552449-1563837	*SURF4*	Surfeit gene 4
18	Chr3:1565031-1572802	*SURF2*	Surfeit gene 2
19	Chr3:1573382-1574242	*BBLN*	B-cell lymphoma 6
20	Chr3:1580662-1603234	*CIZ1*	CIZ1 zinc finger protein
21	Chr3:1779052-1818833	*GOLGA2*	Golgin subfamily A member 2
22	Chr3:1826866-1837222	*PLA2G4F*	Phospholipase A2 group IV F
23	Chr3:1848158-1869130	*PLA2G4C*	Phospholipase A2 group IV C

Supplementary Table S10: Genes Located 500 kb Upstream and Downstream of the *P. csikii* Candidate Sex-associated region on Chromosome 3

Table S10. Significantly enriched GO terms among genes located near the candidate sex-associated region in *P. csikii* (Chr3: 14.92 Mb ± 500 kb). Shown are GO terms with nominal $p < 0.01$, the number of genes overlapping each term, and the associated genes.

GO Term ID	Description	Gene Count	Gene Symbols	p-value
GO:0140694	non-membrane-bounded organelle assembly	4	*MYL2, HAUS4, GOLGA2, PLA2G4C*	0.0009627
GO:0006650	glycerophospholipid metabolic process	3	*MTMR3, PLA2G4F, PLA2G4C*	0.0042001
GO:0051235	maintenance of location	3	*HSPA5, CIZ1, PLA2G4C*	0.0065666
GO:0006644	phospholipid metabolic process	3	*MTMR3, PLA2G4F, PLA2G4C*	0.0079383
GO:0046486	glycerolipid metabolic process	3	*MTMR3, PLA2G4F, PLA2G4C*	0.0088372
GO:1903894	regulation of IRE1-mediated unfolded protein response	2	*HSPA5, DDRGK1*	0.0001065
GO:1900102	negative regulation of ER unfolded protein response	2	*HSPA5, DDRGK1*	0.0001228
GO:0036498	IRE1-mediated unfolded protein response	2	*HSPA5, DDRGK1*	0.0002214
GO:1900101	regulation of ER unfolded protein response	2	*HSPA5, DDRGK1*	0.0005036
GO:0046475	glycerophospholipid catabolic process	2	*PLA2G4F, PLA2G4C*	0.0006477
GO:0072595	maintenance of protein localization in organelle	2	*HSPA5, CIZ1*	0.0009423
GO:1903573	negative regulation of response to ER stress	2	*HSPA5, DDRGK1*	0.0010849
GO:0007020	microtubule nucleation	2	*HAUS4, GOLGA2*	0.0011346
GO:0042149	cellular response to glucose starvation	2	*MTMR3, HSPA5*	0.0013992
GO:0009395	phospholipid catabolic process	2	*PLA2G4F, PLA2G4C*	0.0016301
GO:0032507	maintenance of protein location in cell	2	*HSPA5, CIZ1*	0.0023520

GO:0046503	glycerolipid catabolic process	2	*PLA2G4F, PLA2G4C*	0.0023520
GO:0030968	endoplasmic reticulum unfolded protein response	2	*HSPA5, DDRGK1*	0.0031170
GO:0070972	protein localization to endoplasmic reticulum	2	*HSPA5, DDRGK1*	0.0034514
GO:1905897	regulation of response to ER stress	2	*HSPA5, DDRGK1*	0.0037128
GO:0000281	mitotic cytokinesis	2	*CIT, RAB35*	0.0042625
GO:0034620	cellular response to unfolded protein	2	*HSPA5, DDRGK1*	0.0043576
GO:0045185	maintenance of protein location	2	*HSPA5, CIZ1*	0.0047477
GO:0046785	microtubule polymerization	2	*HAUS4, GOLGA2*	0.0048477
GO:0035967	response to topologically incorrect protein	2	*HSPA5, DDRGK1*	0.0064627
GO:0033559	unsaturated fatty acid metabolic process	2	*PLA2G4F, PLA2G4C*	0.0071685
GO:0061640	cytoskeleton-dependent cytokinesis	2	*CIT, RAB35*	0.0074112
GO:0006690	icosanoid metabolic process	2	*PLA2G4F, PLA2G4C*	0.0084189
GO:0051225	spindle assembly	2	*HAUS4, GOLGA2*	0.0094848
GO:0031109	microtubule polymerization or depolymerization	2	*HAUS4, GOLGA2*	0.0096221

Supplementary Table 11: Significantly Enriched KEGG Pathways for Genes Near the Candidate sex-associated region on chromosome 3 in *P. csikii*

Table S11: Significantly enriched KEGG pathways among genes located near the candidate sex-associated region in *P. csikii* (Chr3: 14.92 Mb ± 500 kb). Shown are pathways with nominal $p < 0.01$, the number of genes overlapping each pathway, and the associated genes.

ID	Description	Count	gene_symbols	p-value
hsa04724	Glutamatergic synapse	4	GNG10, LZTS3, PLA2G4F, PLA2G4C	3.85e-06
hsa04726	Serotonergic synapse	3	GNG10, PLA2G4F, PLA2G4C	1.88e-04
hsa00592	alpha-Linolenic acid metabolism	2	PLA2G4F, PLA2G4C	3.16e-04
hsa00591	Linoleic acid metabolism	2	PLA2G4F, PLA2G4C	4.22e-04
hsa00565	Ether lipid metabolism	2	PLA2G4F, PLA2G4C	0.00118
hsa04913	Ovarian steroidogenesis	2	PLA2G4F, PLA2G4C	0.00127
hsa04014	Ras signalling pathway	3	GNG10, PLA2G4F, PLA2G4C	0.00158
hsa04370	VEGF signalling pathway	2	PLA2G4F, PLA2G4C	0.00169
hsa04730	Long-term depression	2	PLA2G4F, PLA2G4C	0.00169
hsa00590	Arachidonic acid metabolism	2	PLA2G4F, PLA2G4C	0.00186
hsa04664	Fc epsilon RI signalling pathway	2	PLA2G4F, PLA2G4C	0.00223
hsa04912	GnRH signalling pathway	2	PLA2G4F, PLA2G4C	0.00401
hsa04666	Fc gamma R-mediated phagocytosis	2	PLA2G4F, PLA2G4C	0.00454
hsa04750	Inflammatory mediator regulation of TRP channels	2	PLA2G4F, PLA2G4C	0.00454

hsa05231	Choline metabolism in cancer	2	PLA2G4F, PLA2G4C	0.00454
hsa00564	Glycerophospholipid metabolism	2	PLA2G4F, PLA2G4C	0.00490
hsa04611	Platelet activation	2	PLA2G4F, PLA2G4C	0.00726
hsa04270	Vascular smooth muscle contraction	2	PLA2G4F, PLA2G4C	0.00817
hsa04371	Apelin signalling pathway	2	MYL2, GNG10	0.00890
